# Do Lipid-based Nanoparticles Hold Promise for Advancing the Clinical Translation of Anticancer Alkaloids?

**DOI:** 10.3390/cancers13215346

**Published:** 2021-10-25

**Authors:** Jian Sheng Loh, Li Kar Stella Tan, Wai Leng Lee, Long Chiau Ming, Chee Wun How, Jhi Biau Foo, Nurolaini Kifli, Bey Hing Goh, Yong Sze Ong

**Affiliations:** 1 School of Pharmacy, Monash University Malaysia, Bandar Sunway, Subang Jaya 47500, Malaysia; loh.jiansheng@monash.edu (J.S.L.); How.CheeWun@monash.edu (C.W.H.); 2 School of Pharmacy, Faculty of Health & Medical Sciences, Taylor’s University, Jalan Taylors 1, Subang Jaya 47500, Malaysia; stellalikar.tan@sd.taylors.edu.my (L.K.S.T.); jhibiau.foo@taylors.edu.my (J.B.F.); 3 School of Science, Monash University Malaysia, Subang Jaya 47500, Malaysia; lee.wai.leng@monash.edu; 4 PAP Rashidah Sa’adatul Bolkiah Institute of Health Sciences, Universiti Brunei Darussalam, Gadong BE1410, Brunei; long.ming@ubd.edu.bn (L.C.M.); nurolaini.kifli@ubd.edu.bn (N.K.); 5 Health and Well-Being Cluster, Global Asia in the 21st Century (GA21) Platform, Monash University Malaysia, Subang Jaya 47500, Malaysia; 6 Centre for Drug Discovery and Molecular Pharmacology (CDDMP), Faculty of Health & Medical Sciences, Taylor’s University, Jalan Taylors 1, Subang Jaya 47500, Malaysia; 7 Biofunctional Molecule Exploratory Research Group (BMEX), School of Pharmacy, Monash University Malaysia, Subang Jaya 47500, Malaysia; 8 College of Pharmaceutical Sciences, Zhejiang University, Hangzhou 310058, China

**Keywords:** alkaloid, anticancer, lipid-based nanoparticles, liposome, solid lipid nanoparticle, nanostructured lipid carriers

## Abstract

**Simple Summary:**

Alkaloids are natural products that possess numerous pharmacological activities and have been exploited effectively to treat cancer. However, the clinically approved anticancer alkaloids are generally limited by serious side effects due to their lack of specificity to cancer cells, indiscriminate tissue distribution and toxic formulation excipients. Lipid-based nanoparticles represent the most effective drug delivery system concerning clinical translation owing to their unique appealing characteristics for drug delivery. This review aims to assess the potential of different types of lipid-based nanoparticles in encapsulating anticancer alkaloids. Our review shows that alkaloids encapsulated in lipid-based nanoparticles generally displayed an enhanced efficacy and toxicity profile than unencapsulated alkaloids in various cancers. Encapsulated alkaloids also demonstrated the ability to overcome multidrug resistance in cell lines and animal models. These findings support the broad application of lipid-based nanoparticles to encapsulate anticancer alkaloids and facilitate their clinical translation.

**Abstract:**

Since the commercialization of morphine in 1826, numerous alkaloids have been isolated and exploited effectively for the betterment of mankind, including cancer treatment. However, the commercialization of alkaloids as anticancer agents has generally been limited by serious side effects due to their lack of specificity to cancer cells, indiscriminate tissue distribution and toxic formulation excipients. Lipid-based nanoparticles represent the most effective drug delivery system concerning clinical translation owing to their unique, appealing characteristics for drug delivery. To the extent of our knowledge, this is the first review to compile in vitro and in vivo evidence of encapsulating anticancer alkaloids in lipid-based nanoparticles. Alkaloids encapsulated in lipid-based nanoparticles have generally displayed enhanced in vitro cytotoxicity and an improved in vivo efficacy and toxicity profile than free alkaloids in various cancers. Encapsulated alkaloids also demonstrated the ability to overcome multidrug resistance in vitro and in vivo. These findings support the broad application of lipid-based nanoparticles to encapsulate anticancer alkaloids and facilitate their clinical translation. The review then discusses several limitations of the studies analyzed, particularly the discrepancies in reporting the pharmacokinetics, biodistribution and toxicity data. Finally, we conclude with examples of clinically successful encapsulated alkaloids that have received regulatory approval and are undergoing clinical evaluation.

## 1. Introduction

Cancer ranks as the leading cause of morbidity and mortality in the world with an estimated 19.3 million new cases and 9.9 million deaths reported in 2020 [1]. Although stupendous advances have been made in understanding the molecular underpinnings and genomic landscape of cancers, the oncologic outcomes remain poor. Current treatments of cancer include surgery, radiation therapy and chemotherapy [2,3,4]. However, the administration of anti-cancer drugs, including chemotherapeutic drugs, biologic agents and immunotherapeutic drugs using the conventional methods, has been hindered by various pharmacological issues, including toxicities, unsatisfactory therapeutic efficacy and drug resistance [5,6]. These unsatisfactory oncologic outcomes have revitalized the interest in natural product-derived anticancer agents.

Bioactive natural products have been serving as the primary source of medicines by numerous cultures around the world over the past millennia [7]. With the rise of modern scientific approaches, the past century has witnessed a surge of highly active compounds derived from natural products and their derivatives with a precise mode of actions for the treatment of a myriad of diseases. Natural products have gained great interest due to their vast scaffold diversity and structural complexity unrivaled by current synthetic drugs [8]. An analysis of all FDA-approved small-molecule drugs from 1981 to 2014 revealed that approximately 51% were natural products and their derivatives, and about 80% of anti-cancer small-molecule drugs were natural products and their derivatives [9]. Several classes of natural products have been identified, including terpenoid, polyketide, phenylpropanoid and alkaloid [10].

Alkaloid is a class of naturally occurring nitrogen containing heterocyclic organic compounds with a wide range of pharmacological activities, often considered privileged structures in drug discovery [10,11]. Since the commercialization of the first alkaloid morphine in 1826, numerous alkaloids have been isolated and exploited effectively for the betterment of mankind. Today, alkaloid drugs have been approved by the FDA for the treatment of cancer, Alzheimer’s disease, Parkinson’s disease, migraine, pain control, erectile dysfunction, heart failure and many more [12,13]. Alkaloids have demonstrated wide-spectrum anticancer activity by inhibiting topoisomerase I and suppressing microtubule dynamics [14,15]. The most notable anticancer alkaloid drugs that continue to maintain palpable significance in clinical practice include paclitaxel, docetaxel, vincristine, vinblastine, irinotecan and topotecan. However, the administration of anticancer alkaloids has generally been limited by serious side effects due to their lack of specificity to cancer cells, indiscriminate tissue distribution and toxic formulation excipients [16,17,18]. These limitations prompted unceasing investigational efforts to develop effective and safe nanoformulations and improve oncologic outcomes.

Cancer is a disease where the adequacy of delivery of extremely potent yet toxic chemotherapeutic drugs can result in either efficacious responses or serious morbidity [19]. To mitigate these limitations, tailor-designed nanomedicines have emerged as promising strategy for cancer treatment owing to their improved pharmacokinetic properties, therapeutic efficacy, specific targeting of tissues and minimized adverse effects [20]. Furthermore, the use of nanotechnology allows drugs to traverse biological barriers such as the blood-brain barrier [19,21]. Among all the different classes of nanocarriers, lipid-based nanoparticles represent the most established and effective drug delivery system concerning clinical translation, with multiple formulations having already obtained U.S. Food and Drug Administration (FDA) approval for clinical use [20]. Lipid-based nanoparticles have received great attention due to their unique, appealing characteristics for drug delivery, including (1) excellent biocompatibility and biodegradability; (2) improved solubility and stability of difficult-to-deliver drugs, including both hydrophobic and hydrophilic drugs; (3) enhanced therapeutic index by improving efficacy and reducing toxicity; (4) versatility which allows chemical modifications and surface coatings and (5) ability of co-deliver two different anticancer drugs to enable precise spatiotemporal multi-drug treatment [22,23,24,25]. These advantages of lipid-based nanoparticles have been exploited effectively in enhancing the efficacy and reducing the toxicity of anticancer alkaloids, the most exceptional of which are Onivyde (liposomal irinotecan), Marqibo (liposomal vincristine) and Lipusu (liposomal paclitaxel), which have received regulatory approval for clinical use [26]. These uplifting translational successes have motivated an exponential increase in research investigating the potential and effectiveness of encapsulating anticancer alkaloids in different lipid-based nanoparticles. Nevertheless, despite the stupendous therapeutic potential demonstrated by nanomedicines in pre-clinical studies, there are still many shortcomings to be solved.

This review aims to provide some insights and updates on the potential of lipid-based nanoformulations of anticancer alkaloids based on in vitro and in vivo evidence. This review begins with an overview of the current trends and understanding of nanomedicines, then discusses the medicinal uses of alkaloids and their anticancer mechanism of action. Next, the review describes the limitations of currently approved anticancer alkaloid drugs which necessitates their encapsulation in nanoparticles. This is followed by a comprehensive discussion on the most promising lipid-based nanoparticles, which are liposomes, micelles, solid lipid nanoparticles (SLN), nanostructured lipid carriers (NLC) and niosomes. The review then summarizes the findings and limitations of several pre-clinical experiments, focusing on the comparisons of the in vitro and in vivo efficacy and toxicity between free alkaloids and alkaloids encapsulated in lipid-based nanoparticles. Finally, the review highlights several anticancer alkaloids encapsulated in lipid-based nanoparticles that have received regulatory approval and are undergoing clinical evaluation. To the extent of our knowledge, this is the first review to compile the in vitro and in vivo evidence of encapsulating anticancer alkaloids in lipid-based nanoparticles.

## 2. Nanotechnology

The prefix “nano” comes from ancient Greek which represents “dwarf” [27]. One nanometer (nm) is an international System of Units that equals to one billionth of a meter. According to the National Nanotechnology Initiative (NNI), nanotechnology refers to the comprehension and manipulation of matter at atomic or molecular levels between 1 to 100 nm, where unique phenomena facilitate the novel applications [28]. It involves various scientific disciplines (i.e., engineering, technology, medicine, chemistry, physics, biology or a combination of these disciplines) [29]. Nanotechnology has been utilized in numerous medical-related fields including magnetic resonance imaging (MRI), hyperthermic destruction of tumor, proteins detection, diagnosis, pharmacological research, cells and biological molecules purification. Nowadays, numerous nanoparticles have been studied and developed for clinical use including liposomes, nanocapsules, nanorods, nanowires, nanospheres, nanoshells, nanotubes, nanopores and dendrimers [27,30,31].

In general, nanomaterials can be divided into four different categories which are carbon-based, inorganic-based, organic-based and composite-based nanomaterials. Carbon-based nanomaterials can be found in morphologies such as sphere-shaped, ellipsoid-shaped, tube-shaped or horn-shaped [32]. These nanomaterials can further be classified into graphene quantum dots (0-D), carbon nanotubes (1-D) and graphene (2-D) based on their dimensions, where 0-D refers to no dimension, 1-D refers to one dimension and 2-D refers to two dimensions at nanoscale [33,34]. Inorganic-based nanomaterials comprise metal-based and metal oxide-based nanoparticles. Metal-based nanoparticles are comprised of pure metal nanoparticles (i.e., iron, magnesium, zinc, platinum, titanium, copper, gold, silver and alginate nanoparticles). Metal-based nanoparticles can be bound to oxygen, becoming metal oxide nanomaterials (i.e., zinc oxide, silver oxide, etc.) [35,36]. Organic-based nanomaterials are mainly made of organic matter except inorganic and carbon-based nanomaterials. These organic-based nanomaterials can be transformed into liposomes, micelles, dendrimers and polymer nanoparticles that are very useful in drug delivery through noncovalent interactions [36,37]. Composite-based nanomaterials consist of several phases, with one of the phases on a nanoscale dimension that merges different nanoparticles togetherwith enormous or complicated materials, for example hybrid nanofibers and metal-organic frameworks. A composite of these nanomaterials can be a mixture of any polymer, ceramic or metal materials with any organic-based, inorganic-based, metal-based or carbon based nanomaterials [36]. These nanomaterials have potency to revolutionizes the manner at which diseases such as cancer are diagnosed and treated.

### 2.1. Current Trends and Potentials of Nanomedicines

Nanotechnology poses the potential to provide enormous opportunities for advancing medical science over various disciplines. At present, it is used fundamentally due to its importance in most significant inventions in medical treatments. Drug delivery accounts for a considerable portion in nanotechnology advances such as dendrimers, polymeric micelles and liposomes. These drug delivery systems have been used and proven to enhance delivery of various drugs. Disciplines that have gained benefits from nanotechnology advances include drug delivery, surgical treatment, diagnostic imaging and theranostics [26,38,39,40]. Some of the most successful examples of approved nanomedicines include Doxil/Caelyx (liposomal doxorubicin), Abraxane (nanoparticle albumin-bound paclitaxel) and ferumoxytol (iron oxide nanoparticle) [41,42,43].

The application of nanotechnology can be further extended to deliver multiple therapeutic agents in co-loaded nanoparticles, with the most recently approved example being VYXEOS. VYXEOS is a liposomal formulation of daunorubicin and cytarabine at a synergistic molar ratio of 1:5 approved by the FDA for the treatment of acute myeloid leukemia [22]. The pharmacological rationale behind VYXEOS development is to deliver both drugs effectively at the synergistic ratio directly to the leukemic cells, as in vitro and in vivo studies have shown that maximal synergy is achieved when tumor cell lines are exposed to five times as much cytarabine as daunorubicin [44]. However, this efficacious molar ratio could not be adequately achieved and maintained via the traditional 7 + 3 regimen of administering cytarabine and daunorubicin individually, as each drug exhibits suboptimal pharmacokinetics and different rates of metabolism [45]. The clinical success of VYXEOS laid the foundation for more research of nanoparticle-mediated combination therapy to achieve synergistic therapeutic outcomes. In fact, the concept of co-delivery has been extended from combinations of two chemotherapeutic drugs to the co-delivery of chemotherapeutic drugs and gene therapy, immunotherapy, chemosensitizers or imaging agents for maximized therapeutic effects [46,47,48,49,50,51]. Furthermore, the ability to co-deliver therapeutic agents efficiently also proved to be effective in overcoming multidrug resistance (MDR) tumors [52,53]. Interestingly, nanotechnology has also been employed in the development of the BioNTech/Pfizer (BNT162b2) and Moderna (mRNA-1273) COVID-19 mRNA vaccines, which encapsulated the mRNA vaccine within lipid nanoparticles [54,55]. Furthermore, the striking ability of the vaccines to enter clinical trials rapidly, approximately 3 months after obtaining SARS-CoV-2 genome sequences, and the ability to manufacture billions of doses of high-quality vaccine annually further highlight the impressive potential of nanotechnology [56,57].

The continuous improvement of nanotechnology has also introduced new innovations to improve surgical treatments, including cancer surgery. A significant problem in many aggressive cancers, including breast cancer, lung cancer, brain cancer and sarcoma is the presence of minimal residual disease (MRD) despite appearing to achieve complete remission (CR) after initial treatment. MRD is defined as cancer persisting in a patient after treatment, and often comprises just tens of cancer cells that cannot be detected by current medical imaging techniques, resulting in increased risk of lethal relapse later [58]. Hormone receptor-positive breast cancer is the typical example of cancer frequently associated with late recurrence, which can occur after 20 years [59]. To remove potential MRD, surgeons routinely resect a large margin of normal tissue surrounding the tumor, which increases surgical morbidity. To address this issue, a laser pulse-activated nanoevent, called the plasmonic nanobubble, was developed to provide real-time intraoperative in vivo detection of MRD to guide precise and selective surgical resection of MRD with minimal damage to adjacent normal tissues [60]. Plasmonic nanobubbles are bubbles generated when metallic nanostructures convert light energy into highly localized heat that overheats liquid in their proximity, leading to a liquid-vapor phase transition and formation of vapor nanobubbles [61]. While this innovative nanotechnology has yet to be tested clinically, this unique on-demand threshold-activated transient nanoevent offers a new avenue for investigation in other applications, including diagnostics, therapy and theranostics.

### 2.2. Application of Nanotechnology in Drug Delivery

#### 2.2.1. Improved Bioavailability

Drug administration through the oral route has historically been the preferred route of administration, especially in chronic illnesses which require repeated dosing, as it is simple, convenient, economical and non-invasive, thus contributing to the greatest degree of patient compliance [62,63]. However, effective oral drug delivery is often limited by various obstacles, including physicochemical barriers, biopharmaceutical barriers, physiological barriers and clinical barriers [64]. Many bioactive molecules from the natural sources have a high molecular size, resulting in a poor ability to cross the lipid membrane and poor absorption capacity, ultimately leading to reduced bioavailability and efficacy [65]. As such, several nanotechnology-based systems have been applied to improve the bioavailability of oral drugs. Among all the different nanoformulations, lipid-based nanoformulations have shown immense promise due to their ability to enhance the stability, solubility and permeability in the gastrointestinal tract [66,67]. Lipid nanocarriers were shown to enhance the oral bioavailability of hydrophilic and hydrophobic drugs in animals, including anticancer drugs [68,69,70,71], antiviral drugs [72,73], cardiovascular drugs [74], central nervous system drugs [75,76] and peptides [77]. Despite these promising in vivo results, several obstacles remain to be addressed, particularly understanding the mechanism of lipid-based nanoparticles absorption in order to overcome the bottleneck to the development of oral nanomedicines [66].

#### 2.2.2. Passive and Active Targeting

A central paradigm behind intensive research on cancer nanomedicines has been the concept of preferential extravasation and the accumulation of nanoparticles within the tumor interstitium, a phenomenon described as the enhanced permeability and retention (EPR) effect [78]. This concept is justified by the observation that the inter-endothelial gaps in the tumor blood vessels formed during disorganized angiogenesis have a range of up to 2000 nm [79]. In addition, the EPR effect also relates to the findings that solid tumours tend to have a deficient functional intratumoral lymphatic system to remove extravasated nanoparticles [80]. Therefore, scientists are exploiting the EPR effect in the design of nanoparticles to induce passive transport and the accumulation of drugs in the tumor. Studies have reported that up to 10–15% of injected nanoparticles accumulate at the tumor site in vivo, as compared to 0.1% of the injected free drug, which may be attributed to the EPR effect [81,82].

However, this predominant concept is being increasingly challenged by the nanotechnology community recently due to the poor clinical translation of nanomedicines after three decades of research [83]. A recent study addressed this question by performing imaging techniques and computational analysis to study the transport mechanism of nanoparticles into solid tumors. The study found that the frequency of inter-endothelial gaps is far less abundant to account for the nanoparticle accumulation in the tumor. The study reported that nanoparticles enter tumors mainly through active transcytosis by endothelial cells [84]. Furthermore, a meta-analysis of preclinical studies published over the past ten years revealed that only a median of 0.7% of nanoparticles’ injected dose (% ID) reaches the tumors in mice [82]. However, it is important to recognize the limitations of these studies and exercise caution in drawing general conclusions that de-emphasize the EPR effect, as the study by Sindhwani and colleagues only studied the extravasation of one type of nanoparticle at three sizes (15 nm, 50 nm and 100 nm of colloidal gold). Besides, Wilhelm and colleagues evaluated the effectiveness of nanomedicines based on an unconventional parameter (% ID) and did not consider traditional pharmacokinetic parameters such as peak drug concentration (C_max_), clearance (CL), elimination half-life (*t*_1/2_) and volume of distribution (V_d_) which are regularly used to govern regulatory approval of drugs [85]. Nevertheless, Sindhwani and colleagues provided compelling evidence that active transcytosis may act as the dominant transport mechanism of nanoparticles into tumors and motivates researchers to revisit the long-established paradigm and understand the mechanism governing extravasation.

Moreover, EPR effects are highly heterogenous and have been shown to vary over time during tumor development, differing substantially between animal models and patients, among different tumors from the same origin, and among tumors within the same individual [86]. The heterogeneity of EPR effects is the result of the heterogenous nature of intratumoral blood flow, variable vascular permeability, lymphatic drainage network, interstitial tissue pressure, density and composition of the extracellular matrix (ECM) [87,88,89]. These intrinsic heterogeneities of tumors in patients were observed primarily in large clinical tumors than in smaller tumours and have greatly impeded successful clinical translation of nanomedicines [90]. Thus, it may be inappropriate to draw conclusions that underestimate the potential of EPR effects in nanomedicines before understanding the interactions that influence the fate of nanoparticles in biological systems. In fact, the strategy of applying EPR-effect enhancers has been increasingly discussed, including physical methods and pharmacological agents to induce vessel permeabilization, normalization, disruption and promotion [86,90,91,92,93]. The importance of patient stratification has also been highlighted, recognizing that clinical trials of patients with high or low levels of EPR will lead to vastly different therapeutic outcomes [31,86]. Recent studies have employed ferumoxytol-enhanced magnetic resonance imaging (MRI) to quantify and characterize tumor-EPR heterogeneity, where higher levels of ferumoxytol accumulation are correlated with greater lesion size reduction following Onivyde treatment [94,95]. Furthermore, the pessimistic conclusions that EPR effects are absent in humans may be due to poorly designed nanoparticles, particularly those with a low plasma half-life (t_1/2_) or active pharmaceutical ingredients (API) that are readily converted free low molecular weight drugs [91]. In contrast to passive targeting, active targeting involves grafting the nanoparticle surface with specific targeting ligands, including antibodies, antibody fragments, peptides, aptamers and folic acid to direct the nanoparticles to the receptors or antigens expressed at the cancer cell surfaces and enhance tumor accumulation [96,97,98,99,100,101]. This requires an extensive understanding of the specific characteristics of the tumor site and receptor structures of the targeted disease cells. These targeting ligands facilitate selective internalization of nanoparticles into cancer cells via selective receptor-mediated endocytosis, allowing enhancement of cytotoxic activity while avoiding adverse effects on non-cancerous tissues [102,103]. Some of the most widely used ligands in cancer nanomedicines include transferrin, hyaluronic acid, folic acid and arginine-glycine-aspartate (RGD) peptide [104,105,106]. Furthermore, surface functionalization of nanoparticles with targeting ligands also endows them with the ability to traverse physiological barriers such as intestinal mucosa and the blood-brain barrier [26,107,108,109]. However, it is important to understand that targeted nanoparticles still rely on EPR effects to extravasate and reach the tumor cells [93]. This may be the reason why targeted nanoparticles do not radically improve the biodistribution of nanomedicines, and active targeting is often seen as a complementary approach to EPR to improve the efficacy of nanomedicines [78]. In fact, studies comparing the accumulation of nanoparticles in tumors did not observe significant difference between EPR-mediated passive targeting and peptide-mediated or transferrin-mediated active targeting [110,111,112]. Despite the advantages reported for active targeting, only limited actively targeted nanoparticles have reached clinical trials, and none have advanced past clinical trials [83]. Some examples of promising targeted nanomedicines in clinical trials include the BIND-014 prostate-specific membrane antigen (PSMA)-targeted docetaxel nanoparticle for metastatic prostate cancer (Phase 2 completed) [113,114], SGT-53 anti-transferrin receptor single chain antibody fragment (anti-TfR-scFv) liposomal nanoparticle delivering wild-type p53 gene for advanced solid tumor (Phase 2 ongoing; NCT02340117) [115], SGT-94 anti-TfR-scFv liposomal nanoparticle delivering RB-94 gene for metastatic genitourinary cancer (phase 1 completed) [116] and anti-epidermal growth factor receptor (anti-EGFR) immunoliposomes loaded with doxorubicin (anti-EGFR ILs-dox) for advanced solid tumor (phase 2 ongoing; NCT02833766) [117]. Another very promising approach of targeted drug delivery involves magnetic targeting to target drugs precisely to desired tumor regions with the aid of magnetic nanoparticles guided by the external magnetic field [118,119]. Interestingly, the lipid-based nanocarriers showed enhanced radiation and chemotherapy-induced immunogenic cell death as well [120].

**Figure 1:**
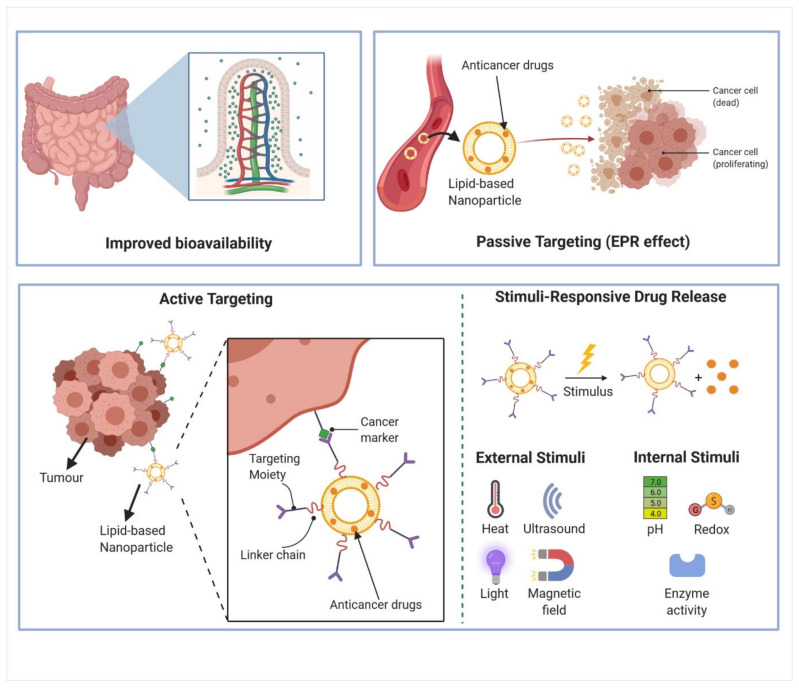
Advantages of nanoparticles in drug delivery. The nanosized drugs demonstrated immense promise due to their ability to enhance the stability, solubility and permeability in the gastrointestinal tract. Apart from the passive targeting by enhanced permeability and retention effect, the nanoparticles can also be formulated by targeting moiety such as an antibody to enhance the drug targeting. External and internal stimuli could also be employed to trigger the drug release at the specific site and specific time, thereby enhancing the drug-targeting properties.

With these aforementioned benefits and innovations provided by nanotechnology in drug delivery (Figure 1), numerous opportunities are presented to improve the biomedical applications of natural products, which are generally characterized by low aqueous solubility and chemical instability that severely hinder their formulations into parenteral drugs. Furthermore, a considerable portion of natural products have poor intestinal permeability and bioavailability which limit their development into oral drugs [121]. In fact, the alkaloids such as paclitaxel and docetaxel are categorized as Class IV drugs (low solubility-low permeability) under the Biopharmaceutics Classification System (BCS) [122]. The detailed limitations of alkaloids are discussed in Section 3.2. These limitations, together with the rapid emergence of targeted therapies, have led to the marginalization of anticancer natural products by pharmaceutical companies [123]. However, the exciting advances in nanotechnology for drug delivery have spurred new opportunities to reformulate natural products and rekindled interest in anticancer natural products, especially alkaloid compounds.

## 3. Alkaloid

Alkaloids are ubiquitous in nature. They are mostly found in plants, and can also be produced by terrestrial animals, marine organisms, microorganisms such as bacteria, fungi and insects. Approximately 20% of plant species contain alkaloids, most of which are biosynthetically derived from amino acids lysine (Lys), ornithine (Orn), tryptophan (Trp), tyrosine (Tyr) and phenylalanine (Phe) [124].

Alkaloid is a class of naturally occurring heterocyclic organic compounds that contain a nitrogen atom. With over 20,000 structurally characterized members, alkaloids remain one of the most medicinally important classes of compounds with a wide range of pharmacological activities, often considered privileged structures in drug discovery [10,11]. In fact, the first naturally derived pure medicine was morphine, an alkaloid isolated from opium poppy in 1805 and commercialized by Merck in 1826 [13]. Since then, numerous alkaloids have been isolated and exploited effectively for the betterment of mankind.

Due to its vast structural diversity and widespread distribution in nature, several classification systems have been used to classify alkaloids, including chemical classification, taxonomic classification, pharmacological classification and biosynthetic classification, each with their own strengths and limitations. In this review, we have adopted the chemical classification to classify the alkaloids based on their chemical structures as this is the most established classification scheme for alkaloids. On this classification basis, the main classes of alkaloids in our review are indole, quinoline, isoquinoline, pyrrolidine, pyridine, piperidine, tropane, indolizidine, terpenoid, purine, imidazole and steroidal alkaloids. Table 1 summarizes different types of medicinally significant alkaloids according to the chemical classification.

Today, alkaloid drugs have been approved by the FDA for the treatment of cancer, Alzheimer’s disease, Parkinson’s disease, migraine, pain control, erectile dysfunction, heart failure and many more [12]. Alkaloids have been widely utilized in various solid tumors and hematological malignancies as a monotherapy or in combination with other chemotherapeutic drugs [125,126,127,128,129]. Thus, much effort has been devoted to elucidate and decipher the mechanism of action of these anticancer alkaloids.

### 3.1. Anti-Cancer Properties of Alkaloids

The remarkable progress in understanding the nature of cancer has allowed us to rationalize the vast complexity of cancer pathogenesis and identify a few major hallmarks of cancer acquired by practically all lethal cancers [130,131,132]. Several alkaloids have demonstrated excellent activity in targeting these cancer hallmarks and are currently being investigated in preclinical studies, and some have successfully entered the clinics as chemotherapeutic drugs. The most notable anticancer alkaloids that continue to maintain palpable significance in clinical practice include paclitaxel, docetaxel, vincristine, vinblastine, irinotecan and topotecan.

Alkaloids have demonstrated the ability to eradicate cancer cells by overcoming their ability to sustain proliferation and evade apoptosis by suppressing microtubules dynamics. Microtubules are cytoskeletal filaments that permeate the cytoplasm of all eukaryotic cells and play a pivotal role in numerous biological processes, including intracellular transport, cell motility, cell morphology maintenance and formation of mitotic spindle to facilitate chromosome separation during cell division [133,134,135,136,137]. Microtubules are inherently dynamic and capable of organizing and restructuring into different architectures in precise timing and location to facilitate various cellular functions [138,139]. During mitosis, the microtubule dynamics are increased by 20–100-fold [140]. As such, microtubules are important targets for anticancer drugs. Natural products targeting microtubules, including alkaloids, have demonstrated remarkable effectiveness in the treatment of both solid tumors and hematological malignancies [141]. Microtubule-targeting agents are broadly classified into microtubule-stabilizing agents (taxanes) and microtubule-destabilizing agents (vinca alkaloids) [15].

Taxanes such as paclitaxel and docetaxel are microtubule-targeting agents that work by stabilizing microtubule dynamics to inhibit cell entry into mitosis. They bind to β-tubulin and promote the polymerization of microtubules to form stable microtubules, resulting in the disruption of microtubule dynamics [142,143,144]. By interfering with microtubule dynamics, paclitaxel and docetaxel inhibit mitotic spindle formation, arrest cancer cells in the metaphase of mitosis, and eventually trigger apoptotic cell death through mitotic catastrophe [15,145]. Taxane-induced mitotic spindle defect induces chronic activation of the spindle assembly checkpoint (SAC) to arrest the metaphase-to-anaphase transition until chromosomes are properly attached and aligned to the spindle microtubules [146]. Chronic SAC activation inhibits ubiquitination and proteasomal degradation of cyclin B1 and chronically increases the activity of CDK1, resulting in sustained mitotic arrest in the G2/M phase [147,148].

However, during mitotic catastrophe, a shift of the cellular response from mitotic arrest to apoptosis occurred through upregulation of the tumor necrosis factor (TNF)-like cytokine 1A (TL1A) that activates death receptor 3 (DR3). This subsequently initiates the recruitment of FADD and caspase-8 to form the death-inducing signaling complex (DISC), and activates the executioner caspases (caspase-3/-7) to execute extrinsic apoptosis [145]. Prolonged mitotic arrest due to persistent activation of SAC following taxanes treatment also leads to cell death via the intrinsic apoptosis pathway, which is regulated by the B cell lymphoma-2 (BCL-2) family [149,150,151]. Taxanes accumulate pro-apoptotic signals and inactivate anti-apoptotic proteins, resulting in the activation of BCL-2 effector proteins BAX and BAK. BAX and BAK oligomerize at the mitochondria and cause MOMP, inducing release of cytochrome-*c* that promotes the formation of apoptosomes. This subsequently leads to the engagement of caspase-9, ultimately the activation of caspase-3 and caspase-7 [152,153]. Paclitaxel has been reported to bind directly to BCL-2 in mitochondria and induce apoptosis [154]. A recent study reported that preventing SAC silencing through p31^comet^ depletion enhanced paclitaxel-mediated apoptosis and markedly potentiated the cytotoxicity of paclitaxel [155].

Another major class of the microtubule-targeting agent is the microtubule-destabilizing agents. Similar to taxanes, vinca alkaloids (vincristine, vinblastine, vinorelbine, vindesine, vinflunine) are remarkable anticancer drugs that target β-tubulin [156]. However, instead of promoting the polymerization of microtubules to disrupt microtubule dynamics, vinca alkaloids bind to a different site of β-tubulin to depolymerize the microtubules [157]. Vinca alkaloids bind between two tubulin heterodimers near the exchangeable GTP site to inhibit the GTP to GDP hydrolysis and GDP-GTP exchange. This triggers a conformational change in tubulin heterodimers from straightened conformation favored for polymerization to curved conformation, lowering the amount of tubulin available for polymerization. This microtubule dynamics disruption prevents mitotic spindle from assembling normally and causes mitotic arrest at the metaphase [157,158,159]. Similar to taxanes, prolonged mitotic arrest caused by vinca alkaloids triggers mitotic catastrophe and induction of apoptosis through the intrinsic and extrinsic pathways in cancer cells [145,160,161].

On the other hand, irinotecan and topotecan are highly potent semi-synthetic analogues of alkaloid camptothecin which have demonstrated wide-spectrum anticancer activity by inhibiting topoisomerase I and inducing catastrophic DNA damage [14]. DNA topoisomerases are ubiquitous and complex vital enzymes responsible for regulating fundamental DNA transactions such as replication, transcription, recombination and repair. Topological problems such as disordered DNA entanglements and knots may be generated during these DNA transactions which, if left unresolved, can lead to genomic instability. Topoisomerase I resolves these topological constraints that arise from RNA polymerase II activity by producing transient single-stranded nick, relaxing the strand and re-ligating the double-stranded DNA structure [162,163,164]. Topoisomerase I inhibitors irinotecan and topotecan stabilize and trap the topoisomerase I-DNA-cleaved complexes (TOP1cc), disabling the re-ligation of the nicked strand and preventing the release of topoisomerase. The trapped TOP1cc eventually collides with advancing replication forks, resulting in lethal and irreversible double-strand breaks and ultimately causes cancer cell death [164,165].

In response to DNA double-strand breaks induced by topoisomerase I inhibitors, the DNA insults are sensed by the MRE11–RAD50–NBS1 (MRN) complex which promotes the activation of ataxia-telangiectasia mutated (ATM) kinase. ATM checkpoint signaling phosphorylates and activates checkpoint kinase 2 (Chk2), leading to cell cycle regulation. ATM together with Chk2 also phosphorylates p53, reducing p53 affinity to its negative regulator E3 ubiquitin ligase MDM2, and resulting in p53 stabilization. Once p53 is activated and stabilized, it acts as a transcription factor and induces the expression of genes involved in cell cycle arrest (p21) and apoptosis (Bax, PUMA, NOXA). This leads to the activation of protease activity of caspases of both intrinsic and extrinsic apoptosis pathways (caspase-8, caspase-9 and caspase-3/7) [166,167,168]. The MRN activation also induces activation of ataxia–telangiectasia and RAD3-related (ATR) kinase which phosphorylates checkpoint kinase 1 (Chk1), arresting the cell cycle in the S phase [169,170].

### 3.2. Limitations of Current Alkaloid Anticancer Drugs Formulation

Despite all the unique anticancer mechanism of actions demonstrated by alkaloids via modulation of several pathways, the full potential of currently available alkaloids and the commercialization of potential anticancer alkaloids could not be achieved due to their lack of specificity to cancer cells, indiscriminate tissue distribution, dose-limiting side effects and toxic formulation excipients [16,17,18,171]. This section summarizes the limitations of taxanes (paclitaxel and docetaxel), vinca alkaloid (vincristine) and topoisomerase I inhibitor (irinotecan) to provide an overview of the challenges faced by these approved drugs and to provide insights into the obstacles possibly holding back the successful commercialization of promising alkaloids.

#### 3.2.1. Taxanes

Paclitaxel is a highly hydrophobic chemotherapeutic drug that can cause embolism when injected intravenously without co-solubilizers due to the presence of particulate drug matters [172]. To enhance its water solubility and enable parenteral administration, paclitaxel is formulated in a 1:1 (*v*/*v*) mixture of polyethoxylated castor oil (Cremophor EL) and dehydrated ethanol as the vehicle [173]. The amount of Cremophor EL administered is about 25 mL at the recommended dose of 175 mg/m^2^ once every three weeks, which necessitates a long infusion duration [174]. However, the use of Cremophor EL as solubilizing agents is clinically associated with several severe side effects, including anaphylaxis and life-threatening hypersensitivity reactions even with corticosteroid and antihistamines premedication [175,176]. Approximately 30% of patients receiving paclitaxel without premedication have been reported to experience these hypersensitivity reactions [18]. Cremophor EL has also been reported to cause nephrotoxicity and neurotoxicity [177]. Furthermore, studies have also shown that Cremophor EL adversely affects the efficacy of paclitaxel due to its ability to form plasma micelles capable of entrapping paclitaxel [178]. This leads to a reduced volume of distribution, increased systemic drug exposure and reduced drug clearance, resulting in the non-linear pharmacokinetics of paclitaxel observed clinically [177,179]. The fraction of drug trapped in micellar bodies is made unavailable to tumor sites despite increasing the dose, as higher doses introduce a higher concentration of Cremophor EL, further limiting the bioavailability and anti-tumor activity [177]. The non-linear pharmacokinetics, unpredictable activity and toxicity profile of paclitaxel further complicate combination chemotherapy regimens.

Similar to paclitaxel, the second generation taxane docetaxel is a hydrophobic drug with low aqueous solubility. To improve its solubility and enable intravenous administration, docetaxel is formulated with non-ionic surfactant polysorbate 80 (Tween 80) and ethanol, where the former is associated with hypersensitivity reactions [180]. Approximately 30% of patients receiving docetaxel without premedication have been reported to experience these hypersensitivity reactions, and 2% of patients experience severe reactions with premedication [18]. Although docetaxel possesses linear pharmacokinetics, there is large interindividual pharmacokinetics variability, particularly clearance and area under the curve (AUC), which causes highly unpredictable efficacy and toxicity profiles [181]. The most prominent toxicity of docetaxel is its hematological toxicities such as neutropenia, which correlates with the systemic exposure to unbound drugs [182]. All these aforementioned limitations prompted unceasing investigational efforts to develop novel nanoformulations for taxanes and obviate the need for surfactants including Cremophor EL and polysorbate 80.

#### 3.2.2. Vinca Alkaloids

As vinca alkaloids target cancer cells exclusively during metaphase, it is ideal to increase the drug concentration at the tumor site for a prolonged duration to kill actively dividing cancer cells during the most sensitive part of their cell cycle [183]. However, this could not be achieved due to their rapid plasma clearance and dose-limiting side effects such as sensory and motor peripheral neuropathies, which are the most common and severe in vincristine treatment [184,185]. As a result, the approved adult dose of vincristine is 1.4 mg/m^2^ and routinely capped at 2 mg to prevent severe peripheral neuropathy, resulting in underdosing in patients with a body surface area larger than 1.43 m^2^ [16]. This suboptimal dosing is significant, as a study found that the average body surface area of 3613 adult cancer patients was 1.79 m^2^, with 1.91 m^2^ for men and 1.71 m^2^ for women [186]. This indicates that nearly all adult patients receiving vincristine are greatly underdosed, leading to unsatisfactory treatment outcomes. To overcome these pharmacokinetics and dosing limitations, liposomal vincristine (Marqibo) was developed and received accelerated FDA approval in 2012 for the treatment of Philadelphia chromosome (Ph)-negative acute lymphoblastic leukemia at the dose of 2.25 mg/m^2^ without dose capping [187,188]. As compared to free vincristine, Marqibo has prolonged plasma circulation, lower clearance and higher AUC without apparent toxicity exacerbation at doses unachievable by free vincristine [187,189,190,191].

#### 3.2.3. Topoisomerase I Inhibitors

Irinotecan has a complex metabolism due to the involvement of various drug-metabolizing enzymes, such as cytochrome P450 (CYP) and uridine diphosphate glucuronosyltransferase 1A (UGT1A), and thus is subjected to large interindividual pharmacokinetic variabilities. Following administration, irinotecan is metabolized by carboxylesterases to its active metabolite, SN-38, which is approximately 100- to 1000-fold more potent than the parent drug. SN-38 is then immediately inactivated within minutes via glucuronidation by *UGT1A1* to the inactive SN-38 glucuronide (SN-38G) and excreted via the bile, resulting in the short half-life of irinotecan [192]. The use of irinotecan is often limited by dose-limiting toxicities, such as diarrhea and neutropenia, which shows significant interindividual variability even at standard doses due to genetic variations in drug-metabolizing enzymes and drug transporters [17]. Compounding these limitations is the fact that enterohepatic recirculation of SN-38G exposes SN-38G to bacterial enzymes in the intestines which convert SN-38G to active SN-38, resulting in serious and life-threatening late diarrhea among patients receiving irinotecan [171]. The strategy of *UGT1A1* genotype-guided irinotecan dosing has been recently investigated in a phase III trial which reported a significant increased pathological complete response (pCR) rate when combined with capecitabine-based neoadjuvant chemoradiotherapy in patients with locally advanced rectal cancer [193]. To overcome the limitations of irinotecan, several approaches have been investigated. The most successful example is the nanomedicine liposomal irinotecan (Onivyde) which was approved by the FDA in 2015 for the treatment of metastatic pancreatic cancer in combination with 5-fluorouracil and folinic acid. Nanoliposomal irinotecan encapsulates and prevents irinotecan from being converted into SN-38 in the circulation to increase and sustain the intra-tumoral levels of both irinotecan and SN-38 [194,195].

## 4. Lipid-Based Nanoparticles for Encapsulation of Anticancer Alkaloids

**Figure 2:**
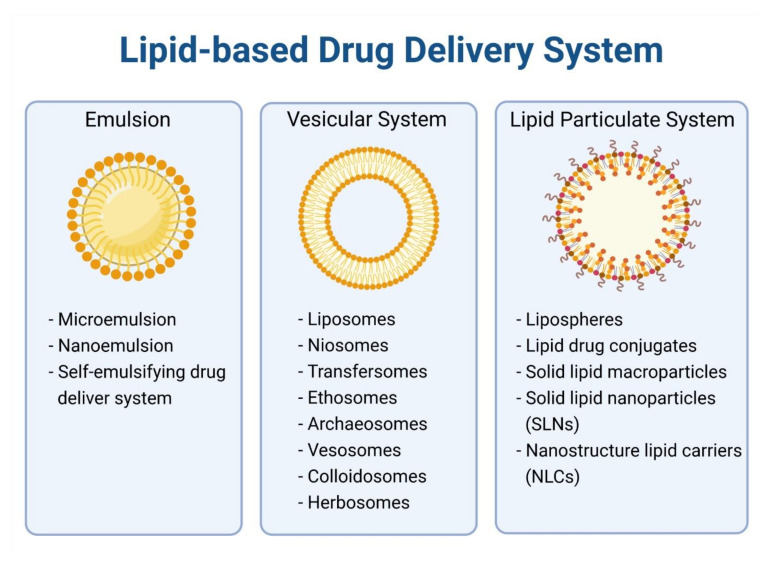
Classification of Lipid-based Nanocarriers.

Considering all the foregoing hints, nanocarriers could be a potential strategy to overcome the limitations of alkaloids. Nanocarriers are around 5 to 200 nm in size and can be used in a wide range of applications [196]. They can be categorized into various types (e.g., organic, inorganic, polymeric, biological, lipid-based nanocarriers) according to their physical properties, chemical properties, morphology and size. Among the carriers, lipid-based nanocarriers offer an alternative to solubilize, encapsulate and deliver alkaloids in a programmed manner to enhance their water solubility, bioavailability and anticancer efficacy [25]. Examples of lipid-based nanocarriers are liposomes, solid lipid nanoparticles (SLN) and nanostructured lipid-carriers (NLC) (Figure 2). These drug carriers are made up of biocompatible lipids triglycerides, cholesterol and phospholipids in which most of them are derivatized based on or extracted from natural sources, resulting in their excellent biodegradability and biocompatibility [197]. Excipients use in lipid carriers such as cholesterol, PEG and phosphatidylcholine have established toxicology data and safety profiles for their use in pharmaceutical products, further strengthening their potential as the ideal drug delivery system [198]. Lipid-based nanoparticles with an average size of 100 nm and have longer circulation half-lives which enhance their propensity to extravasate through vascular fenestrations of tumors’ vasculature, thus enhancing the potency of anticancer agents [199]. However, it is relatively difficult to prepare such small-sized lipid-based nanoparticles. In this regard, a “top down” size reduction approach that requires high energy input (e.g., sonication) and a “bottom up” method that produces nanoparticles by lipid condensation from solution can be used to solve this problem [200]. As far as we know, clinically approved cancer nanomedicines that utilize lipid-based nanocarriers as drug delivery agents have particle sizes larger than 80 nm, for example, Doxil80–100 nm), Marqibo (100 nm) and Abraxane (130 nm) [190,201,202]. Numerous studies have been conducted to encapsulate alkaloids into lipid nanocarriers.

### 4.1. Liposome

Liposomes are one of the extensively studied lipid vesicles, which are made up of phospholipids and an aqueous medium. Lipid vesicles are formed when lipids interact with the aqueous medium, where the lipid hydrophilic head group envelops the aqueous core along with exposure of the hydrophilic tail group to the external medium. Owing to this distinct structural property, drugs can be entrapped in the lipid bilayer or loaded in the internal aqueous core of liposomes depending on their hydrophilicity, for example, Doxil and Onivyde [203]. The first nano-based formulations approved by the FDA for cancer treatment are liposomal anticancer drugs. Doxil was the first doxorubicin-loaded liposomal drug which received FDA approval in 1995 to be used in the treatment of AIDS-related multiple myeloma and Kaposi sarcoma due to its lower cardiotoxicity and higher efficacy as compared to free doxorubicin alone. The clinical success of Doxil further established the potential of liposomes in drug delivery, and many promising liposomal formulations are currently being scrutinized intensively in clinical studies [203,204]. Reduced dose-limiting toxicities, improved undesirable pharmacokinetics and drug solubility are the primary objectives in liposomal development to deliver alkaloids. Liposomes’ stability is one of the major issues in the development of liposomal alkaloids [197]. The drug to lipid ratio and lipid composition have to be taken into consideration in preparing physically stable liposomal alkaloids. Neutral zwitterionic lipids are the most commonly used lipids in preparing liposomes such as phosphatidylcholine. Phospholipids and cholesterol can be added to enhance stability, reduce aggregation and improve permeability of drugs [205,206]. Introduction of the PEGylation approach in the early nineties was shown to prolong the circulation time of liposome significantly due to steric stabilization of vesicles [207]. Likewise, incorporation of PEG-modified lipids was found to enhance delivery and cytotoxicity of paclitaxel liposome to human cancer cells [208]. However, PEG, which is hydrophobic in nature, was later found to lower the polarity of the aqueous matrix and destabilize the liposomes, leading to rapid drug leakage. To overcome the issue, poly(zwitterionic) polymers such as poly[2-(methacryloyloxy)ethyl phosphorylcholine) (PMPC) [209] and poly(carboxybetaine) (PCB) [210] were introduced to replace PEG. That said, liposomes have disadvantages including limited drug loading capacity due to space available in liposomal lipid membranes, inadequate control of drug release, reproducibility issues and stability issues [211]. The drug-loading capacity of liposome can be solved by a remote loading approach where the drug will be added to preformed liposomes via pH gradient or ion gradient competent to create a pH gradient [212].

In 2014, liposomes were used to co-encapsulate doxorubicin and a contrast agent (Magnevist) or hyperthermic agent (Fe_3_O_4_) [213,214]. Doxorubicin has also been encapsulated into ultrasound-sensitive liposomes in which the drug release can be facilitated by sonication that degrades liposomes, co-encapsulated with curcumin in liposomes to enhance anti-tumor efficacy of doxorubicin in the C26 colon cancer cell line, encapsulated in arginine-penetrating peptides/PEG modified liposomes to decrease in vitro cytotoxicity and enhance delivery of doxorubicin for the treatment of ovarian cancer [215,216,217]. In addition, incorporation of docetaxel in anacardic acid and PEG-modified liposomes have proven to stabilize docetaxel [218]. Many different liposomes and modified liposomes were designed to carry a variety of anticancer agents, such as paclitaxel and 5-fluorouracil [219].

Liposomal preparation requires a cryogenic atmosphere brought on to the introduction of vesicular drug delivery systems using non-ionic surfactants. It was named niosome consisting of either uni- or multi- lamellar vesicles. Niosome was first introduced in the cosmetic industry and its potential application in drug delivery was only discovered thenceforth [220]. The unique structure of niosome allows it to encapsulate both hydrophilic materials in vesicular aqueous core and lipophilic materials in the bilayer domain. It is composed of non-ionic surfactants with cholesterol and can increase the size and provide charge to vesicles, therefore, enhancing the entrapment efficiency of noisome. Niosome has a similar structure as liposomes and it is expected to be a better delivery system than liposomes due to its stability, cost and entrapment efficiency [221,222]. Nevertheless, only few niosome formulations are tested in clinical trials, and no formulations are commercially marketed heretofore owing to their low efficacy [223]. Ethosome is also a modified version of classical liposomes and is mainly composed of phospholipids, ethanol and water in which the concentration of ethanol is relatively high, differentiating it from other vesicular carriers. High ethanol constituents of ethosome improve skin permeability by releasing the encapsulated materials into deeper layers and systemic circulation [224,225]. For transdermal delivery of drugs, ethosome is superior over classical liposomes due to its higher entrapment efficiency, more negative zeta potential and smaller size [226,227]. Despite having these superiorities, only few ethosome formulations enter clinical trials due to limitations such as low yield and suitability to carry potent drugs only but not drugs which require a high concentration of blood [224].

### 4.2. Micelles

Micelles are colloidal systems formed through self-assembly of amphiphilic molecules. They can be further classified into polymeric micelles, lipid micelles and lipid polymeric hybrid micelles based on types of amphiphilic molecules. Amphiphilic molecules in lipid micelles are normally small-molecule surfactants. Unlike liposomes having a lipid bilayer, lipid micelles have a monolayer with an outer hydrophilic corona enclosing an inner hydrophobic core form by hydrophobic acyl chains [228]. Critical micelle concentration (CMC) refers to concentration of surfactants above which micelles form. It is an important surfactants parameter to consider in designing micelles [197]. Enhancing solubility of drugs is the primary objective of designing micelles as drug nano-carriers. Hydrophobic drugs such as docetaxel and paclitaxel are carried in the lipophilic core of micelles [228]. Nevertheless, lipid micelles possess major limitations: unstable in bloodstream as dissociation occurred upon dilution below the micelle forming concentration (CMC) and limited interior hydrophobic space influencing the loading capacity of drugs. In order to stabilize the micelles by reinforcing weak intermolecular interactions, several strategies such as the formation of covalent crosslinking (for i.e., shell crosslinking) and non-covalent crosslinking (for i.e., diblock copolymers) can be used [229]. Lipid micelle has been used to deliver various drugs including paclitaxel, doxorubicin and camptothecin in the preclinical stage [230,231,232]. Cabral and co-workers identified that small polymeric micelles are suitable in delivering drug to the tumor site due to their favorable size range between 30 to 100 nm which penetrates well in highly permeable tumors [200]. However, they are not up to the market until today due to insufficient cellular interaction with tumor cells for cellular uptake and poor physical stability in vivo [233]. Even though both micelles and vesicles were formulated in the same principle whereby the lipid molecules reorganized and clustered together in an aqueous solution, the lipid layers were formed differently depending on their shapes. Lipid micelles were formed by wedge-shaped lipid molecules with the hydrophobic tails facing inwards, whereas the vesicles were formed by the cylinder-shaped phospholipid molecules with the hydrophobic tails sandwiched between the hydrophilic head groups [234].

### 4.3. Solid Lipid Nanoparticles

Comparing the lipid-based nanocarriers discussed, solid lipid nanoparticles (SLN) represent a colloidal drug delivery system with an external aqueous phase and internal lipid phase introduced in the early 1990s. They are composed of a combination of various solid lipids such as waxes and fatty acids, as well as mono-, di- and triacylglycerols that form a lipid matrix entrapping drugs or other hydrophobic materials. Beneficial properties of SLN, such as cost effectiveness, non-toxicity, ease of preparation, controlled drug release, good system stability and provision of target-specific effects, made them outstrip other carriers when they were first introduced [235,236]. On top of that, use of IV acceptable solid lipids (e.g., phospholipids, glycerides) and surfactants (e.g., poloxamer 188, lecithin, tween-80) makes SLN a versatile platform for drug delivery readily to translate into clinical application [235]. SLN-based formulations have shown a substantial enhancement in the anti-tumor efficacy of hydrophobic drugs. For example, resveratrol-SLN have greater inhibitory effects against the proliferation, invasion and migration of breast cancer cells than the free drug alone; talazoparib-SLN improve the therapeutic index against triple-negative breast cancer by overcoming homologous recombination-mediated resistance and talazoparib toxicity [237]. However, the highly organized crystalline structure of solid lipids leaves a limited room for drug incorporation which contributes to low drug capacity and drug expulsion during storage [238]. However, the highly organized crystalline structure of solid lipids leaves a limited room for drug incorporation which contributes to a low drug-loading capacity and drug expulsion during storage [238]. To overcome this problem, Muller and colleagues have come out with a novel lipid delivery system called nanostructured lipid carriers (NLC) [239].

### 4.4. Nanostructured Lipid Carriers

NLC are a modified version of conventional SLN by incorporating liquid lipids with a solid lipid. The room between crystal imperfections and fatty acid chains of NLC allows more drug accommodation. Moreover, certain drugs are more soluble in liquid lipids than solid lipids [240]. Hence, NLC as the second generation lipid carriers after SLN reduce drug expulsion during storage and enhance the drug-loading capacity [238]. NLC can be prepared using various surfactants and co-surfactants, allowing them to be formulated for various administration routes (e.g., parenteral, oral, topical, ocular, nasal) to deliver drugs and active substances for biochemical, cosmetic and pharmaceutical purposes [240]. NLC possess favorable properties of low toxicity and controlled drug release, and provide target-specific effects and a high drug load for both hydrophilic and hydrophobic agents, making them outstrip SLN when they were introduced [235,241]. NLC-based formulations have been studied extensively for the delivery of anti-tumor agents. For example, 6-gingerol-NLC has a greater water solubility and oral bioavailability than the free drug alone, penetrating peptides, and hyaluronic-acid-modified artesunate-NLC has a better cell-membrane-penetrating ability against hepatic cancer; thymoquinone-NLC and fluvastatin-NLC have an improved anti-tumor efficacy against hepatic cancer and prostate cancer, respectively [242].

Lipid-based delivery systems attracted a great deal of attention in the past decades as a strategy to enhance anticancer efficacy and overcome delivery barriers and the therapeutic index of various drugs, especially alkaloids. Myriads of preclinical studies have been focused on the use of lipid carriers to deliver alkaloids due to their potential in cancer treatment. Table 2 summarizes alkaloids that have been encapsulated in lipid-based nanocarriers for cancer treatment. Some of them utilized vesicular systems while others used lipid-particulate systems.

## 5. Integration of Lipid-Based Nanoparticles Improves Efficacy and Safety of Alkaloids

### 5.1. In Vitro Efficacy of Alkaloid Encapsulated in Lipid-Based Nanoparticles

The encapsulation of alkaloids in lipid-based nanoparticles have been extensively studied in numerous cancers, such as breast, lung and hepatocellular carcinoma [205]. Table 3 summarizes the in vitro efficacy of encapsulated alkaloids. The encapsulation of alkaloidal drugs in lipid-based nanoparticles generally improves cytotoxicity reflected by a reduction in the IC_50_. Considerable reductions in the IC_50_ were observed in cells treated with encapsulated alkaloids, most notably lipid-based docetaxel, paclitaxel and vincristine as compared to their respective free drugs. The higher anti-cancer effects could be attributed to a possible increase in cellular uptake by modulating active and passive transport to overcome the biological barriers and drug resistance. In addition, differences in the cellular uptake mechanism between the diffusion of free alkaloids and endocytosis of encapsulated alkaloids could be one of the potential contributing factors [274,275,276]. Apart from that, improvements in efficacy were observed in the encapsulated alkaloids ranging from 20.24 to 99.94% as compared to free drugs. Free topotecan exhibited a IC_50_ of 1.88 µg/mL in BT20 breast cancer cells, and the IC_50_ of topotecan-encapsulated liposomes was significantly reduced to 0.33 µg/mL, showing an approximate six-fold reduction compared to the free form of topotecan [266]. Free docetaxel exhibited the IC_50_ of 14.4 µg/mL in MCF-7 cells, whereas liposomal docetaxel exhibited an eight-fold reduced IC_50_ of 1.9 µg/mL with approximately an eight-fold reduction from its free form [243]. Similarly, the IC_50_ of free vincristine and liposomal vincristine in KB cells was 3.47 µg/mL and 0.0021 µg/mL, respectively [260]. In general, alkaloids encapsulated in lipid-based nanoparticles exhibit elevated cytotoxicity at a lower concentration as compared to their respective free drugs. This may be attributed to a smaller nanoparticle size that enhances the drug uptake [26].

Over decades, multidrug resistance (MDR) has been a major complex issue especially in cancer treatment. It can be either intrinsic or acquired due to prolonged drug exposure, resulting in drug efflux from the cancer cells, leading to a reduced intracellular drug concentration and diminished therapeutic efficacy. Increasing the dose of chemotherapeutic agents or using combination therapy are usually the courses of action at this point. However, it might lead to increased patient morbidity as a result of increased toxicity, eventually leading to treatment failure. Moreover, the approach of using combination therapy may be ineffective in preventing the development of drug resistance, as tumors are made of diverse populations of cells. These cells are genetically unstable and will inevitably develop resistance to multiple drugs of the combination therapy [19,278,279]. In fact, the majority of cancer patients died from a disseminated disease that developed resistance to multiple treatment modalities [280]. MDR normally arises from the overexpression of ATP-binding cassette (ABC) transporters, especially ABCB1 (also known as P-glycoprotein or MDR1), ABCG2 (also known as breast cancer resistance protein BCRP) and ABCC1 (also known as multidrug-resistant protein-1 MRP-1) [281]. These ABC transporters have been demonstrated to efflux numerous chemotherapeutic agents, including taxanes, vinca alkaloids, topotecan and irinotecan [280]. Therefore, extensive efforts were undertaken to search for effective treatments to overcome MDR.

Lipid-based nanoparticle encapsulation of chemotherapeutic agents serves as a potential strategy in overcoming MDR. For examples, Yu and co-workers formulated topotecan liposome, and Zhang and co-workers developed paclitaxel-NLC, both tested in MCF-7 and drug-resistant MCF-7/ADR breast cancer cell lines [256,267]. A particularly promising trend was observed in the luminal breast cancer cell line where encapsulated alkaloids displayed a lower IC_50_ value in the resistant cell line compared to the non-resistant breast cancer cell line. In the case of topotecan-liposome, it exhibited a IC_50_ of 2.07 µg/mL in the MCF-7 cell line; however, the IC_50_ was reduced to 0.71 µg/mL in the MCF-7/ADR cell line, showing an approximate three-fold reduction, while for paclitaxel-NLC, it exhibited a IC_50_ of 0.075 µg/mL in the MCF-7 cell line, but the IC_50_ was reduced to 0.065 µg/mL in the MCF-7/ADR cell line. On the other hand, a higher IC_50_ is observed in resistant cell lines; for example, vincristine-liposomes have a IC_50_ value of 0.0021 µg/mL in non-resistant (KB) and 0.33 µg/mL in resistant oral epidermoid (KBv200) cell lines, respectively, while the IC_50_ of paclitaxel-NLC on non-resistant (SKOV3) and resistant ovarian (SKOV3-TR30) cell lines are 0.053 µg/mL and 0.1 µg/mL, respectively [254,256]. Nevertheless, they are still more effective towards oral epidermoid and ovarian cancers when compared to free drugs. This may be attributed to the small particle size of alkaloid-encapsulated lipid nanocarriers which are taken up by cells through endocytosis that may bypass or evade ABC transporters which are responsible to efflux cytotoxic agents after being released into the cytoplasm and the subsequent emergence of MDR in cancer treatment. On top of that, a smaller particle size may increase the intracellular concentration of the alkaloid which will enhance its cytotoxicity in resistant cells [280,282].

### 5.2. In Vivo Efficacy and Toxicity of Alkaloids in Lipid-Based Nanoparticles

The in vivo efficacy and toxicity of the lipid-based nanoparticles are summarized in Table 4. The encapsulation of alkaloid drugs in lipid-based nanocarriers generally improves their anti-tumor efficacies, with better tolerability as reflected by the changes in body weight. In the study by Zhigaltsev and co-workers, the liposomal docetaxel appeared to be less effective than Taxotere (commercial formulation of docetaxel) when administered at the same dose (25 mg/kg). However, they found that the liposomal encapsulation of docetaxel reduces the toxicity of docetaxel and allows a three-fold higher maximum tolerated dose (MTD) than Taxotere, from 29 mg/kg to >88 mg/kg. The increased MTD in liposomal docetaxel may be attributed to reduced systemic exposure to docetaxel and reduced vehicle toxicity due to the absence of surfactant polysorbate 80 [244]. The concept of MTD is fundamental in clinical oncology, where the optimal dose of chemotherapeutic agents is determined based on the safety aspects. The potential for tumor remission is maximized by administering the highest possible dose based on the ability of patients to tolerate the associated side effects [283]. Thus, the ability of lipid-based nanoparticles to increase the MTD of docetaxel by more than three-fold in vivo supports the broad application of nanoparticles in cancer treatment.

Consistent with their in vitro results reporting an enhanced ability to eliminate MDR breast cancer cell lines, Yu and colleagues demonstrated that liposomal topotecan possesses substantially enhanced anti-tumor efficacy in resistant MCF-7/ADR cell xenografts in mice as compared to free topotecan [267]. This is clinically significant, as the ability of nanoparticles to overcome the drug resistance mechanism allows effective use of established chemotherapeutic drugs. Indeed, lipid-based nanocarriers such as liposomes and micelles are the most studied in targeting MDR in cancer [285].

The administration of conventional chemotherapeutic drugs has generally been limited by serious side effects due to their lack of specificity to cancer cells, indiscriminate tissue distribution and toxic formulation excipients. Therefore, it is desirable to develop nanoformulations that possess the ability to target cancer cells passively or actively to minimize the collateral damage towards non-cancerous cells [19,177,180]. As shown in Table 4, considerable reductions in toxicity were observed in animals treated with lipid-based nanoparticles, most notably lipid-based docetaxel, paclitaxel and vincristine, as compared to their respective free drugs. Lipid encapsulation was shown to be able to improve the toxicity profile of the encapsulated drugs by approximately 30% in xenograft mouse models as compared to free drugs, while retaining or even demonstrating superior anti-tumor efficacy. This may be attributed to the EPR effects whereby nanoparticles preferentially extravasate and accumulate in tumor tissues due to the presence of inter-endothelial gaps in the tumor blood vessels formed during disorganized angiogenesis and defective lymphatic drainage [78,80].

It is important to understand that the incorporation of compounds in nanocarriers significantly alters the properties of the compounds and changes the way they interact with biological systems [286,287,288]. However, a portion of the studies summarized in Table 4 did not report the basic toxicity profile, such as the weight changes induced by these lipid-based nanoparticles in animal models. Furthermore, many of these studies did not report the pharmacokinetic and biodistribution data. Previous studies have shown that administered nanoparticles are sequestered by macrophages of the mononuclear phagocyte system (MPS), causing high accumulation in the liver and spleen, resulting in local toxicity in these organs [289,290,291]. Furthermore, pharmacokinetic and biodistribution profiles of nanoparticles are important to predict their anti-tumor efficacy and toxicity profiles [287]. Thus, it is vital to include this information in addition to their anti-tumor efficacy in order to allow unbiased comparisons of lipid-based nanocarriers across experiments and achieve a balance of acceptable toxicity and good efficacy. Uncertainty regarding the hazard and safety information will impede the exploitation and use of these nanoparticles in humans. In fact, many nanomedicines failed in the clinical trials due to unexpected toxicities from preclinical studies, with the most recent example being MM-310 (liposomal docetaxel prodrug). MM-310 was discontinued in 2019 following reports of cumulative peripheral neuropathy in a Phase 1 clinical trial despite demonstrating excellent results in a preclinical study [292,293]. Therefore, it is ideal to standardize evaluation criteria and unify preclinical standards to accelerate clinical translation. Moreover, a number of studies compared their lipid-based nanoparticles with a solution of pure compounds, instead of formulations available on the market, such as Taxol (paclitaxel) or Taxotere (docetaxel). This may generate improper conclusions about the effectiveness and advantages of their nanoparticles as compared to the conventional medicines currently used clinically.

### 5.3. From Bench to Bedside

Similar to conventional drugs, the regulatory approval for nanomedicines is stringent, expensive and time-consuming, requiring evidence to prove their efficacy, safety and quality [294]. Furthermore, successful translation of nanomedicines from bench to bedside are often limited by design complexity which prevents economic production and scale-up production [20]. Nevertheless, a few anticancer alkaloids encapsulated in lipid-based nanoparticles have successfully overcome these hurdles and have been approved by the FDA and foreign equivalents. In this section, we aim to provide a snapshot of anticancer alkaloids encapsulated in lipid-based nanoparticles that have received regulatory approval and the current status of those undergoing different phases of clinical trials (Table 5).

The first FDA-approved anticancer alkaloid that has been successfully encapsulated in lipid-based nanoparticles was liposomal vincristine (Marqibo), which was approved in 2012 for the treatment of Philadelphia chromosome-negative acute lymphoblastic leukemia [45,190]. In the phase I trial that led to its FDA approval, Marqibo was shown to overcome the dosing limitations of vincristine, allowing the administration of vincristine at the dose of 2.25 mg/m^2^ without dose capping and apparent toxicity exacerbation [187,188]. Furthermore, Marqibo has improved pharmacokinetic profiles, demonstrating prolonged plasma circulation, lower clearance and higher AUC than free vincristine [190,191]. A phase I study conducted in children showed that the approved adult weekly dose of 2.25 mg/m^2^ was well tolerated without evidence of neurotoxicity [189].

In 2015, the liposomal irinotecan (Onivyde) received its FDA approval for the treatment of metastatic pancreatic ductal adenocarcinoma (mPDAC) in combination with 5-fluorouracil and folinic acid in patients previously treated with gemcitabine-based treatment. The approval was based on the global phase III NAPOLI-1 trial which reported that the addition of Onivyde significantly improved the median overall survival (OS) compared with 5-fluorouracil + folinic acid alone (6.1 months vs. 4.2 months) (hazard ratio 0.67; *p* = 0.012) with a manageable safety profile in patients who progressed after gemcitabine-based therapy [195]. Subsequent extended follow-up analysis of the long-term survivors of the NAPOLI-1 trial further confirmed the OS advantage of Onivyde + 5-fluorouracil + folinic acid as compared to 5-fluorouracil + folinic acid alone (6.2 months vs. 4.2 months). Approximately 25% patients who received Onivyde + 5-fluorouracil + folinic acid were alive after a one-year follow-up as compared to 13% patients who were given 5-fluorouracil + folinic acid alone [295].

**Figure 3:**
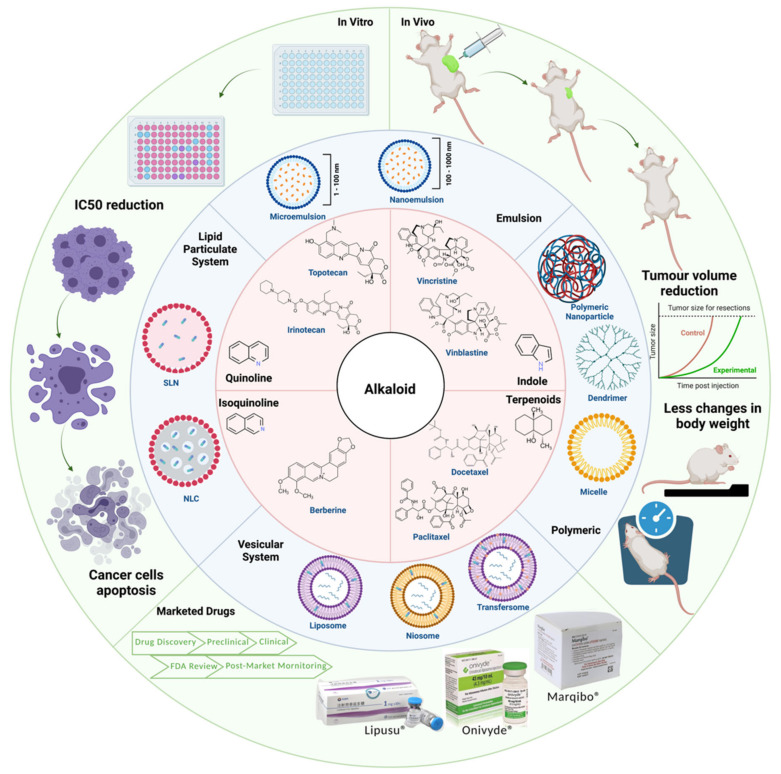
Overview highlighting the types of lipid-based nanoparticles that have been utilized to encapsulate anticancer alkaloids, the in vitro and in vivo anti-tumor effects and products that have successfully received regulatory approval for clinical use. As explored in this review, anticancer alkaloids encapsulated in lipid-based nanoparticles demonstrated enhanced in vitro efficacy (reduced IC_50_), in vivo efficacy (increased tumor volume reduction), in vivo toxicity profile (changes in body weight), as compared to unencapsulated anticancer alkaloids.

On the other hand, Lipusu was the first paclitaxel liposome approved for clinical use by the State Food and Drug Administration of China in 2006. It eliminates the use of the toxic vehicle Cremophor EL, thus reducing the toxicities while retaining the anti-tumor activity of free paclitaxel. A study reported that the hallmark hypersensitivity reactions of paclitaxel was not observed in 53 patients receiving Lipusu-based chemotherapy [296]. In addition, Lipusu has also shown comparable activity as a free paclitaxel but with a lower incidence of adverse effects in metastatic gastric cancer [297]. Randomized phase 4 trials (LIPUSU, NCT02996214) are currently on-going to study the safety and efficacy of the combination of Lipusu and cisplatin as the first-line treatment in patients diagnosed with advanced squamous NSCLC. Figure 3 summarizes the in-vitro, in vivo anti-tumor effects and the anticancer alkaloids encapsulated in lipid-based nanoparticles that have successfully received regulatory approval for clinical use.

## 6. Conclusions and Future Perspectives

In the race against cancer, bioactive natural products such as alkaloids have gained great interest due to their vast scaffold diversity and structural complexity unrivaled by current synthetic drugs [8]. However, the anticancer alkaloids currently employed in clinical practice are largely limited by serious side effects due to their lack of specificity to cancer cells, indiscriminate tissue distribution and toxic formulation excipients. The last few decades have seen considerable progress in nanotechnology, offering a multitude of opportunities and possibilities to overcome these shortcomings. Apart from improving the bioavailability, the drug efficacy was largely improved by the controlled drug release of these nanocarriers [298]. Several stimuli such as temperature [299], pH [300] and redox sensitivity [301] were shown to trigger and enhance the release of the drug cargo in selected locations. Lipid-based nanoparticles, particularly liposomes, have been investigated and exploited in a vast range of pharmaceutical products owing to their unique, appealing characteristics for drug delivery. Lipid-based nanoparticles are envisioned to have a great impact on public health, especially with the recent success of the BioNTech/Pfizer (BNT162b2) and Moderna (mRNA-1273) COVID-19 mRNA vaccine [54,55].

Our review evaluated several lines of in vitro and in vivo evidence of the efficacy and toxicity of anticancer alkaloids encapsulated in lipid-based nanoparticles. Encapsulated alkaloids generally show enhanced in vitro cytotoxicity and an improved in vivo efficacy and toxicity profile than free alkaloids in various cancers. This may be attributed to the superiority in physicochemical properties and pharmacokinetic, pharmacodynamic and biodistribution profiles endowed by lipid-based nanoparticles. Studies also reported the ability of encapsulated alkaloids to overcome the long-dreaded MDR, which is the major cause of treatment failure and deaths among cancer patients [280]. Thus, lipid-based nanoparticles encapsulation proved to be a powerful approach for advancing the clinical translation of anticancer alkaloids. Three liposomal nanoformulations of alkaloids, Onivyde, Marqibo and Lipusu, have been approved for clinical use, with more promising lipid-based nanoparticles currently undergoing different phases of clinical trials. Most of these lipid-based nanoparticles have been designed to exploit the EPR effects to enhance their efficacy and tolerability [86].

With the explosive increase in preclinical studies investigating the effectiveness of nanoparticles, it is important to unify the reporting standards of all the efficacy, toxicity, pharmacokinetics and biodistribution parameters necessary for the nanoscience community to make unbiased evaluations of the effectiveness of these nanoparticles. While a false negative toxicity profile due to the use of poorly predictive preclinical studies may lead to products being potentially harmful to patients, erroneous toxicity signals may also prevent the development of potentially safe drugs. Similarly, overestimation of the efficacy of nanoparticles may put patients participating in clinical trials at risk of ineffective treatments [302,303]. Thus, standardizing the research protocols and reporting criteria will certainly enhance the clinical translatability of nanoparticles. In fact, the FDA has established the Nanotechnology Characterization Lab (NCL) and provided the standardized analytical cascade specifying the key preclinical characterizations as a guide for the nanoscience community to gather the in vitro and in vivo data necessary for Investigational New Drug (IND) applications [83]. However, these discrepancies in reporting standards, particularly the pharmacokinetics, biodistribution and toxicity data, were observed clearly in the studies summarized in Table 4, where a number of studies only reported the efficacy of their nanoparticles.

The number of nanomedicines that have successfully translated to the clinical settings is unsatisfactory, despite a myriad of research articles reporting the superiority of nanoparticles over conventional drugs in preclinical studies. The main reason causing this attrition is the complex heterogeneity in EPR effects, varying over time during tumor development, differing significantly between animal models and humans, among different tumors from the same origin, and among tumors within the same individual [86]. The role and complexity of endocytosis in the uptake of nanoparticles have also been reviewed recently, highlighting the limitations of present experimental methods in understanding the internalization mechanisms of nanoparticles and how they reach the intended site of action [304,305]. Interestingly, a recent study discovered that a threshold dose of 1 trillion nanoparticles overwhelmed the liver and dramatically increased the tumor delivery efficiency of nanoparticles from 0.7% to 12% of the injected dose (% ID) in mice [306]. Moreover, a recent study challenged the long-established EPR effects by providing evidence that the dominant transport mechanism of colloidal gold nanoparticles into solid tumors is through active transcytosis by endothelial cells [84]. Understanding these nanoparticle-biological (nano-bio) interactions will allow us to harness the true potential of nanoparticles and ensure that nano-concepts have a macro-impact in clinical settings.

## Figures and Tables

**Table 1:** Medicinally Significant Alkaloids According to the Chemical Classification.

Class	Drugs	Molecular Formula	Origin	Indication/Uses
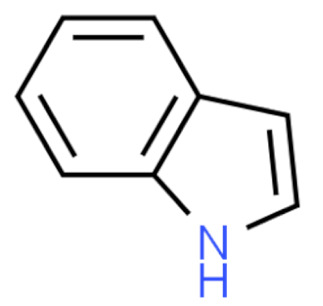 Indole	Vincristine 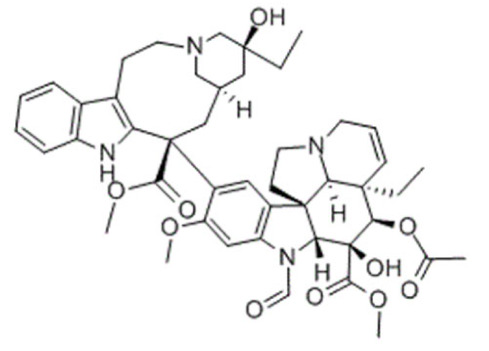	C_46_H_56_N_4_O_10_	*Catharanthus roseus*	Anticancer
	Vinblastine 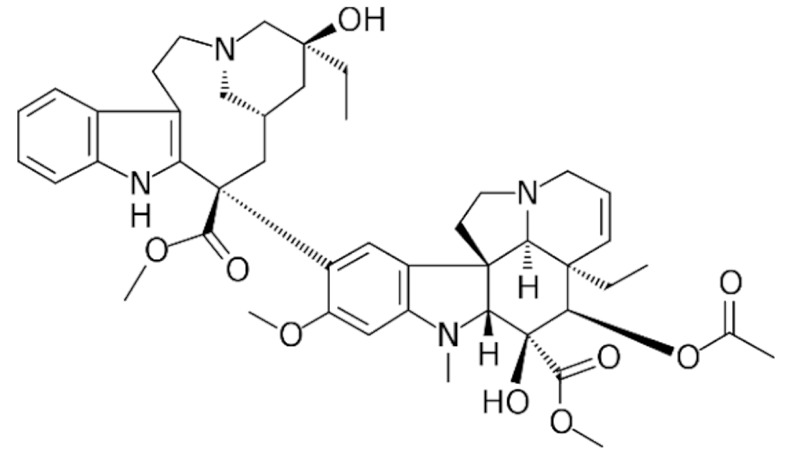	C_46_H_58_N_4_O_9_		
	Vinorelbine 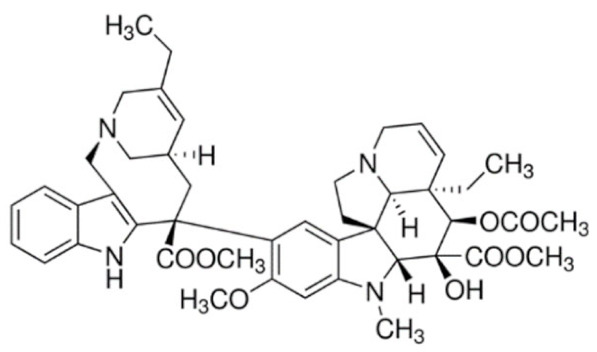	C_45_H_54_N_4_O_8_		
	Vincamine 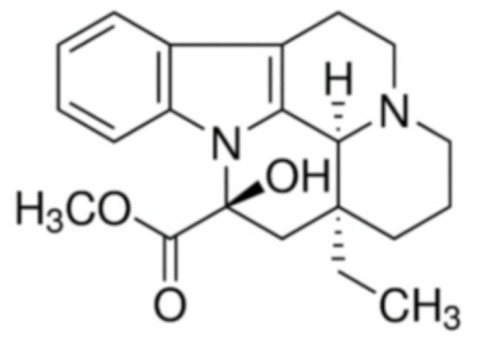	C_21_H_26_N_2_O_3_	*Vinca minor*	Primary degenerative and vascular dementia
	Physostigmine 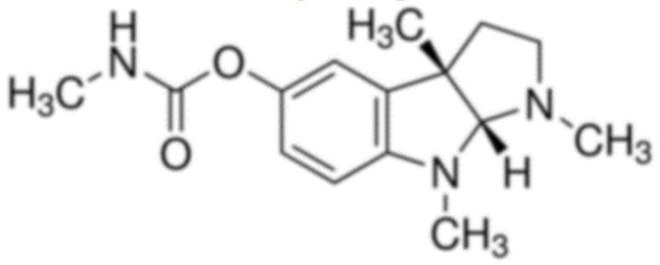	C_15_H_21_N_3_O_2_	*Physostigma venenosum*	Glaucoma
	Ajmaline 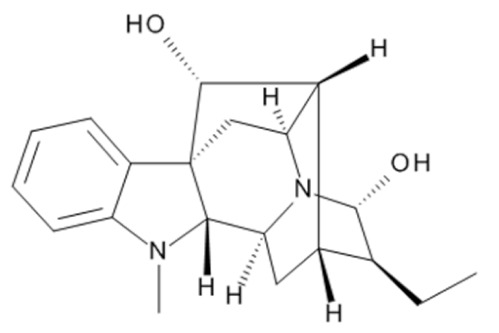	C_20_H_26_N_2_O_2_	*Rauvolfia serpentina*	Anti-arrhythmic
	Ajmalicine 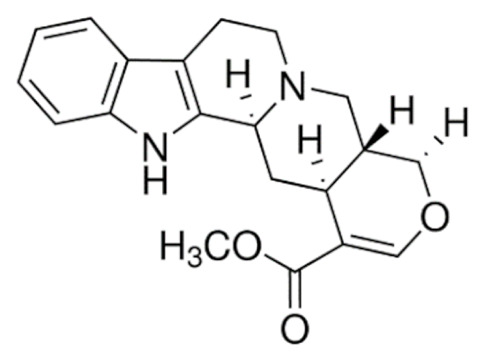	C_21_H_24_N_2_O_3_		Anti-hypertensive
	Reserpine 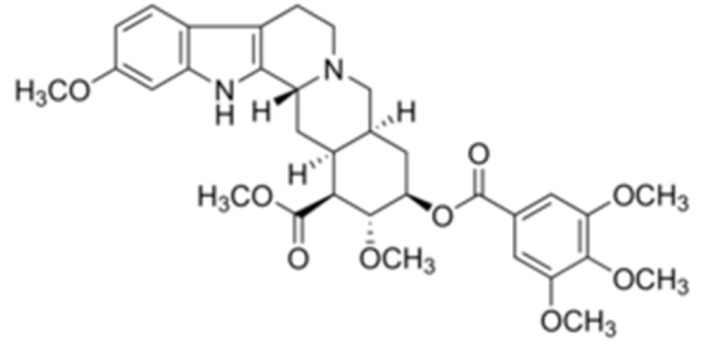	C_33_H_40_N_2_O_9_	*Rauvolfia serpentina*	Anti-hypertensive
	Yohimbine 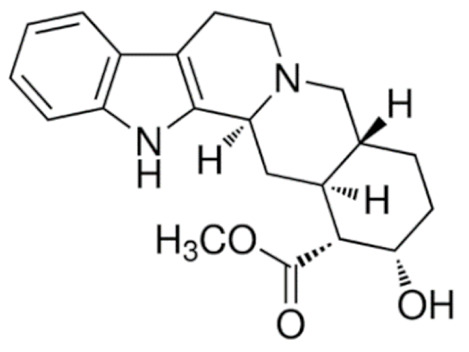	C_21_H_26_N_2_O_3_		Erectile dysfunction
	Strychnine 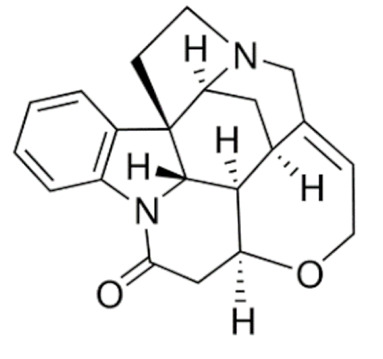	C_21_H_22_N_2_O_2_	*Strychnos nux-vomica*	Convulsant
	Mitragynine 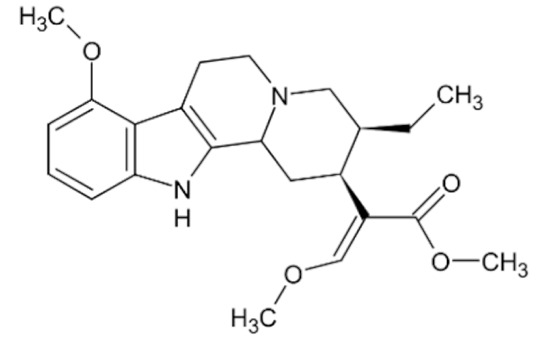	C_23_H_30_N_2_O_4_	*Mitragyna speciosa*	Stimulant, analgesic
	Psilocin 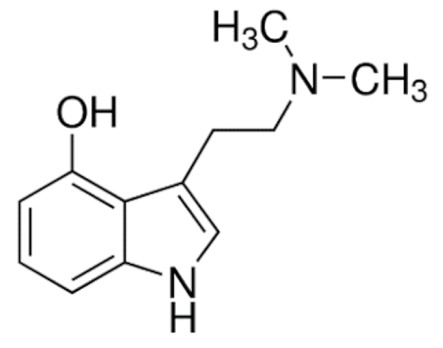	C_12_H_16_N_2_O	*Psilocybe cubensis*	Hallucinogen
	Psilocybin 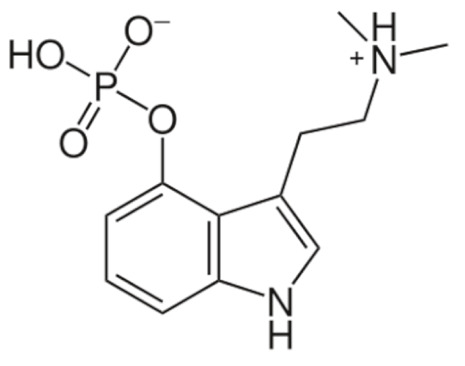	C_12_H_17_N_2_O_4_P		
	Ephedrine 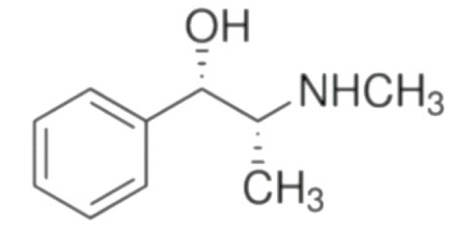	C_6_H_5_CH(OH)CH (CH_3_)NHCH_3_	*Ephedra sinica*	Bronchial asthma
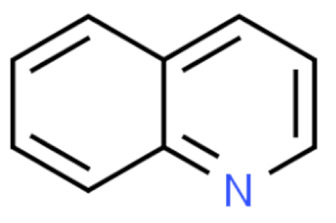 Quinoline	Irinotecan 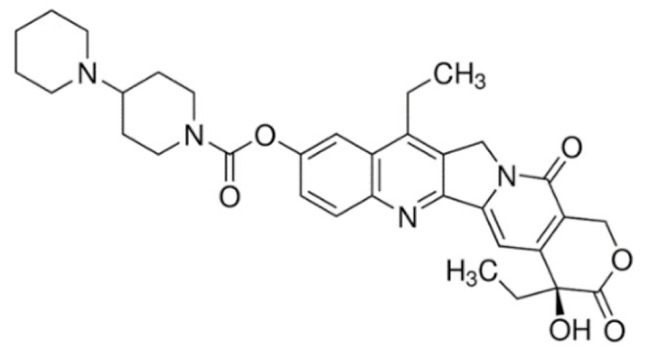	C_33_H_38_N_4_O_6_	*Catharanthus roseus*	Anticancer
	Topotecan 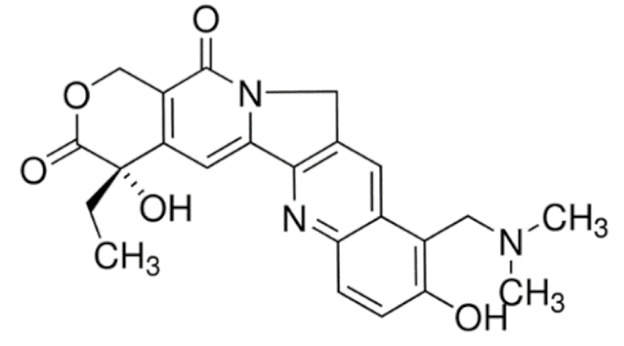	C_23_H_23_N_3_O_5_		
	Colchicine 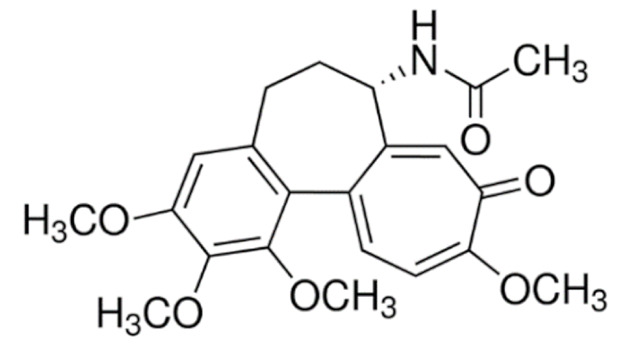	C_22_H_25_NO_6_	*Colchicum autumnale*	Gout
	Quinidine 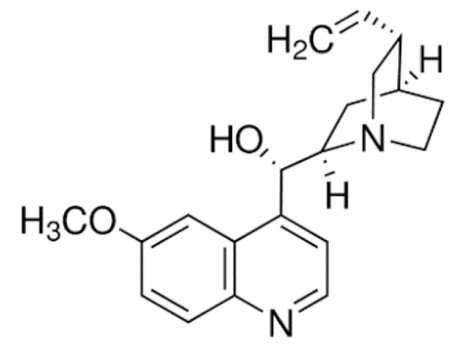	C_20_H_24_N_2_O_2_	*Cinchona officinalis*	Anti-arrhythmic
	Quinine 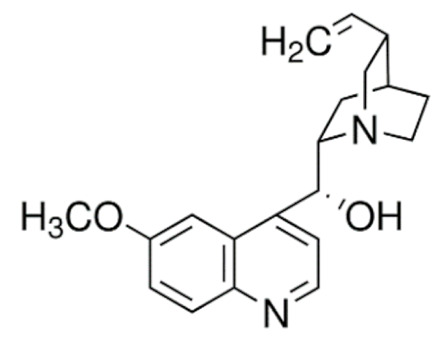	C_20_H_24_N_2_O_2_		Anti-malarial
	Cinchonine 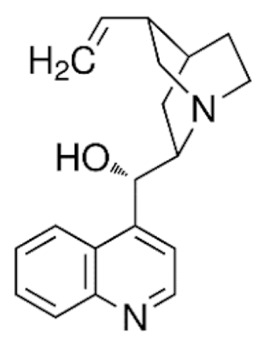	C_19_H_22_N_2_O		
	Cinchonidine 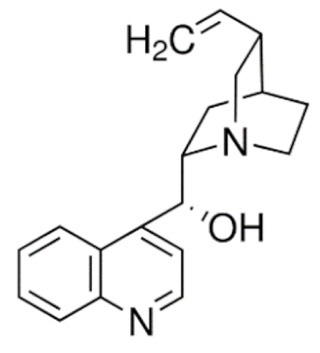	C_19_H_22_N_2_O		
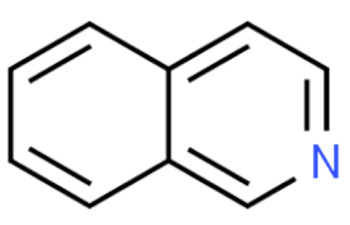 Isoquinolines	Morphine 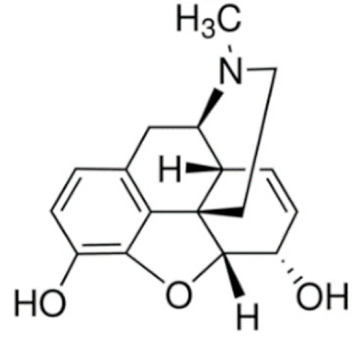	C_34_H_40_N_2_O_10_S	*Papaver somniferum*	Analgesic
	Codeine 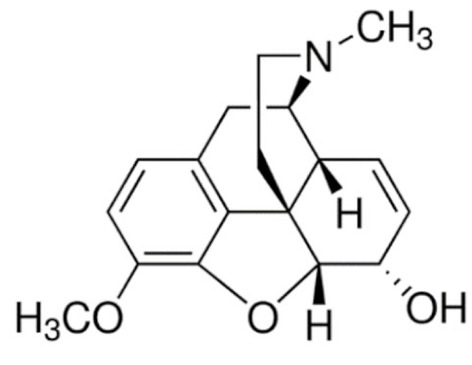	C_18_H_21_NO_3_		
	Heroine 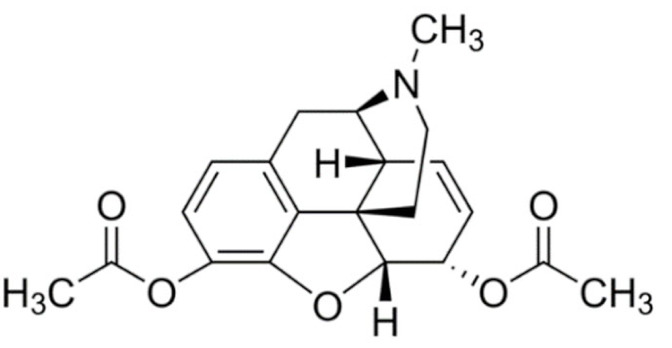	C_21_H_23_NO_5_		
	Apomorphine 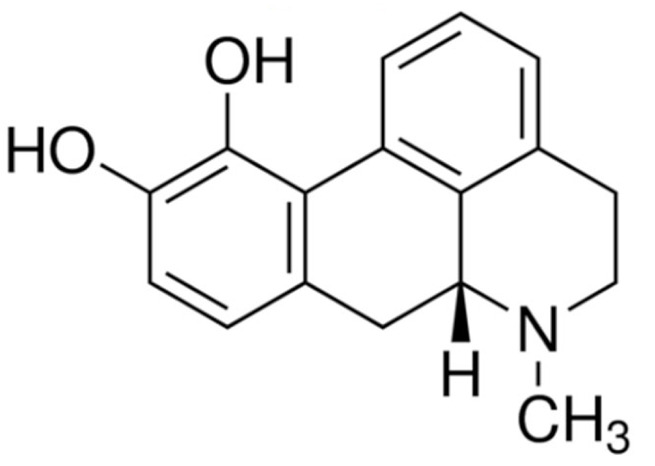	C_17_H_17_NO_2_		Parkinson’s disease
	Noscapine 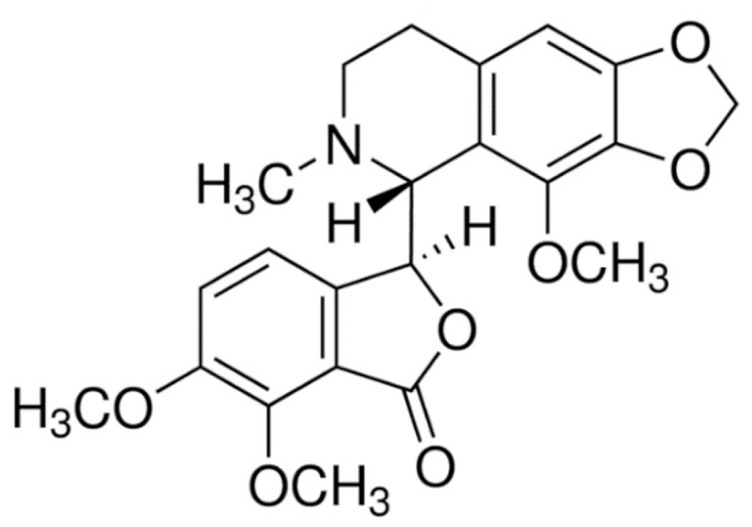	C_22_H_23_NO_7_		Anti-tussive
	Trabectedin 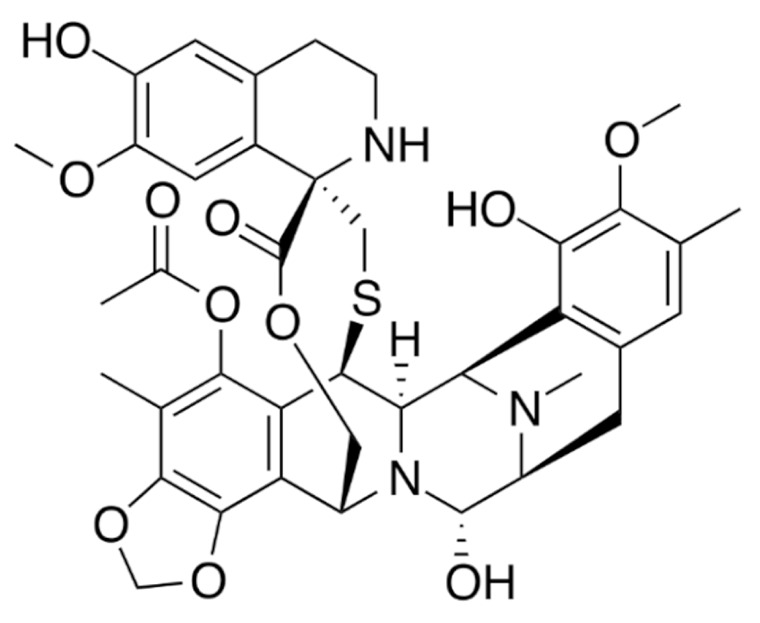	C_39_H_43_N_3_O_11_S	*Ecteinascidia turbinata*	Anticancer
	Berberine 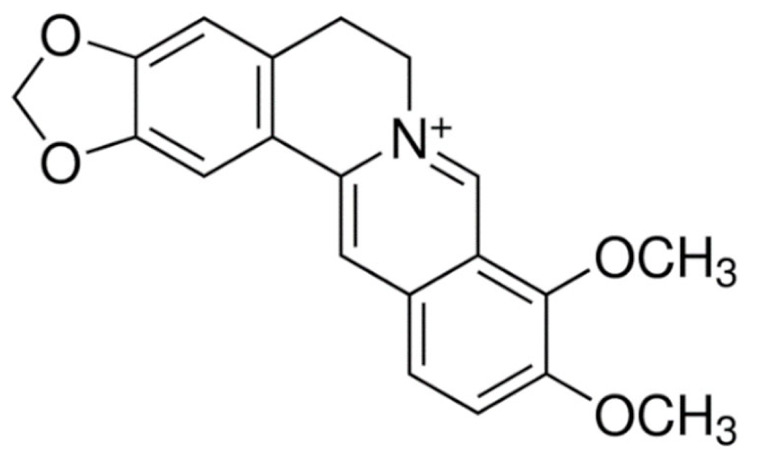	C_20_H_18_ClNO_4_	*Coptis chinensis*	Antimicrobial
	Tubocurarine 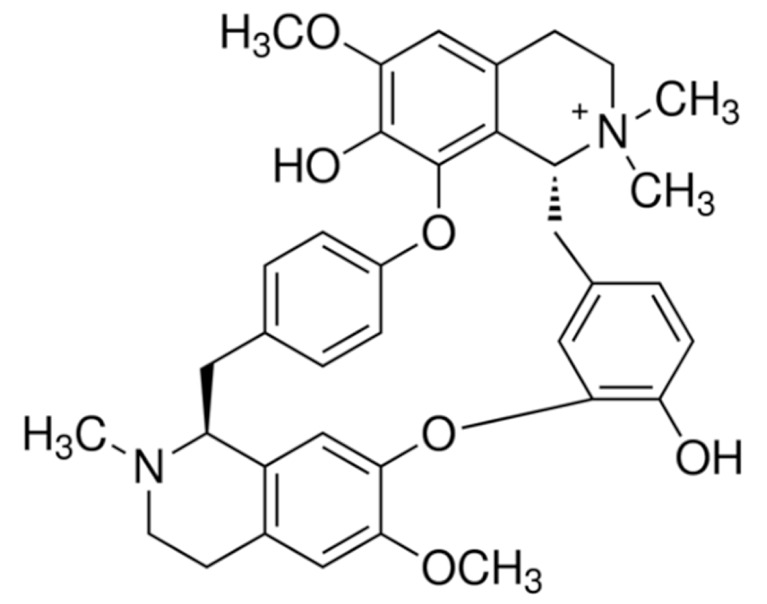	C_37_H_41_ClN_2_O_6_	*Chondrodendron tomentosum*	Skeletal muscle relaxant
	Atracurium 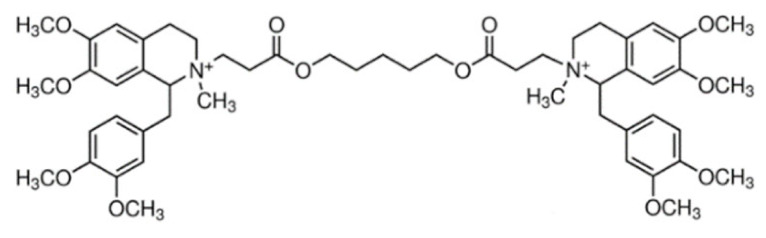	C_53_H_72_N_2_O_12_	*Leontice leontopetalum*	
	Tetrandrine 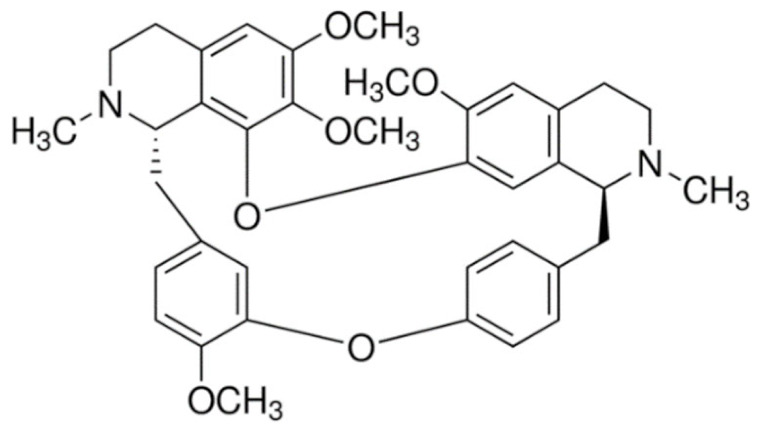	C_38_H_42_N_2_O_6_	*Stephania tetrandra*	Anti-arrhythmic
	Galantamine 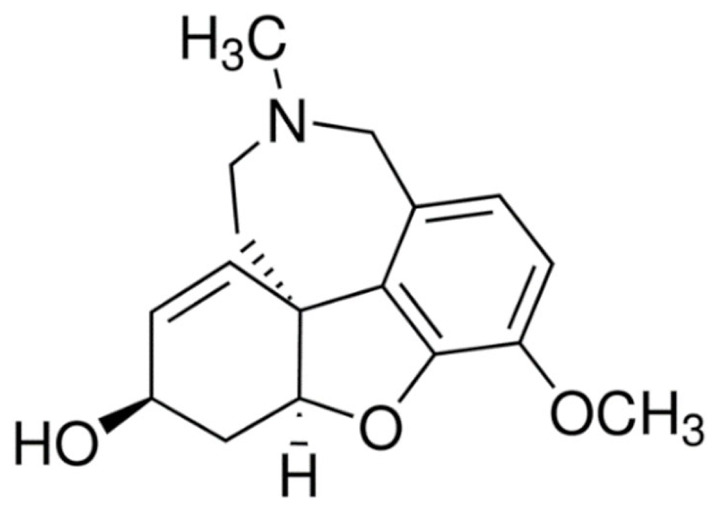	C_17_H_21_NO_3_	*Galanthus nivalis*	Alzheimer’s disease
	Sanguinarine 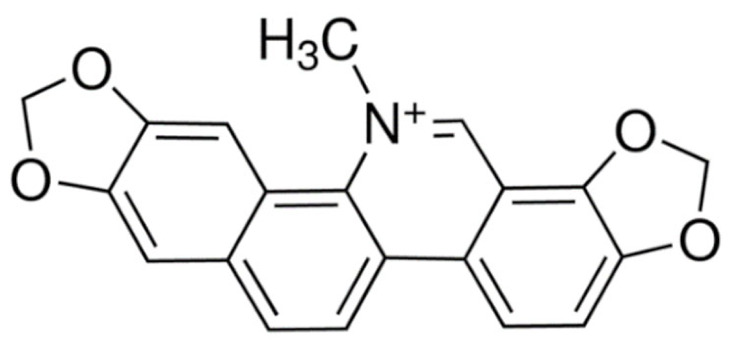	C_20_H_14_NO_4_	*Sanguinaria canadenis*	Antibacterial, antiplaque
	Papaverine 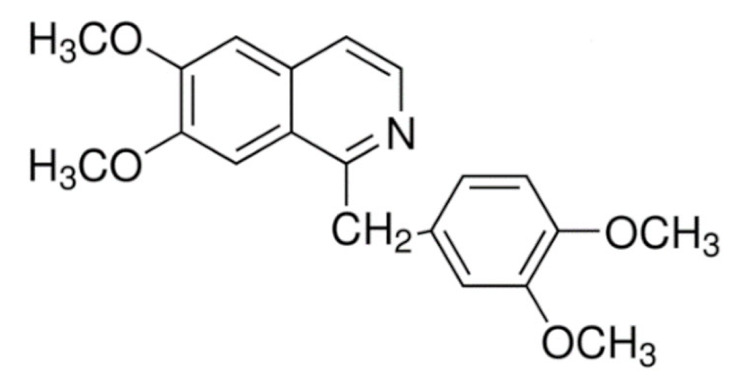	C_20_H_21_NO_4_	*Papaver somniferum*	Vasodilator
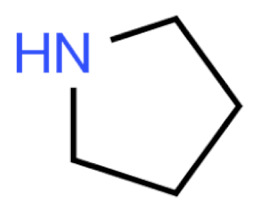 Pyrrolidines	Hygrine 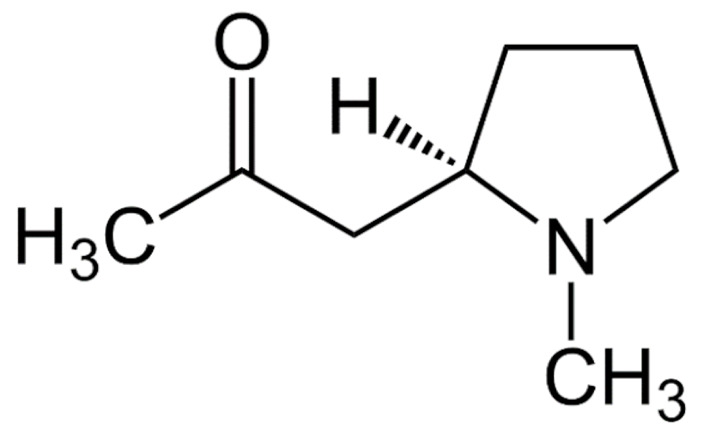	C_8_H_15_NO	*Erythroxylon coca*	Laxative, diuretic
	Cuscohygrine 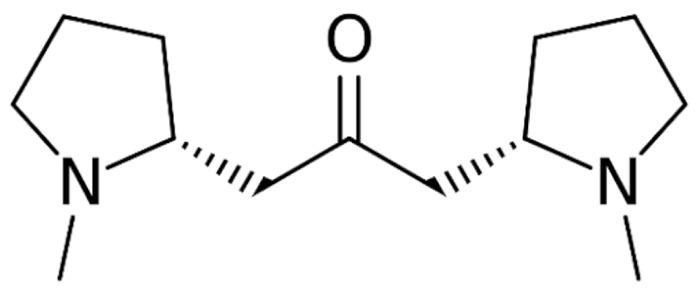	C_13_H_24_N_2_O		
	Stachydrine 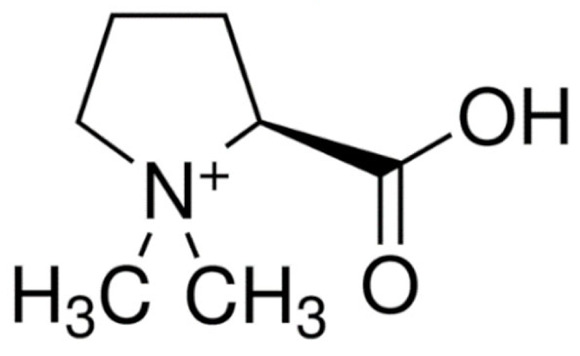	C_7_H_14_NO_2_	*Stachys tuberifera*	Neuroprotectant
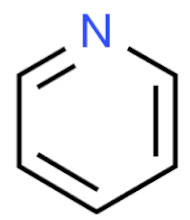 Pyridines	Arecoline 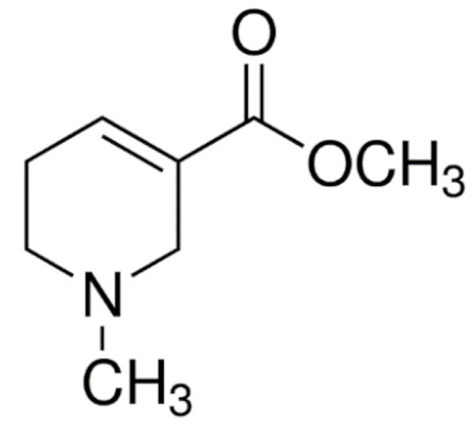	*C_8_H_13_NO_2_*	*Areca catechu*	Muscarinic agonist
	Ricinine 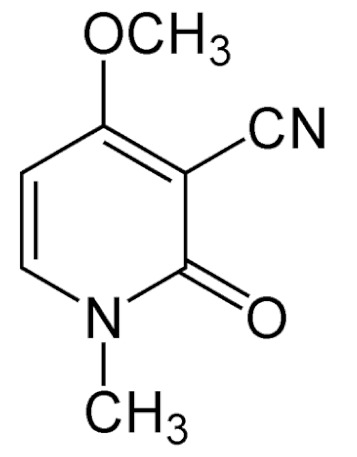	C_8_H_8_N_2_O_2_	*Ricinus communis*	Insecticide
	Trigonelline 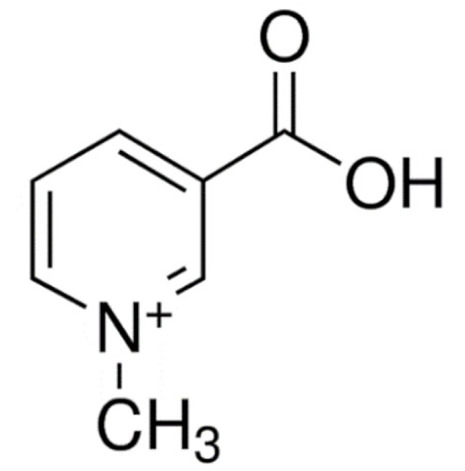	*C_7_H_7_NO_2_*	*Trigonella foenum*-*graecum*	Antidiabetic
	Nicotine 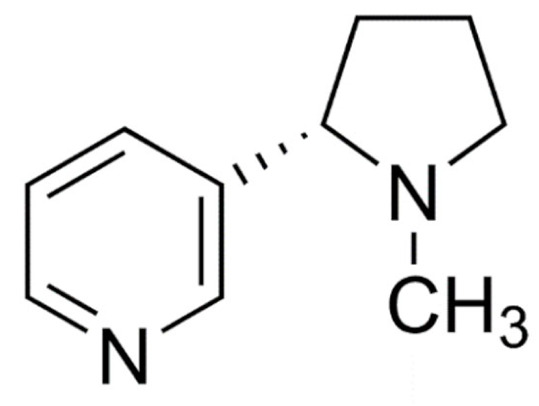	C_10_H_14_N_2_	*Nicotiana tabacum*	Smoking cessation
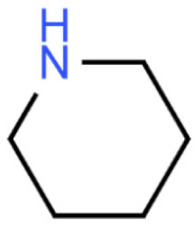 Piperidine	Piperine 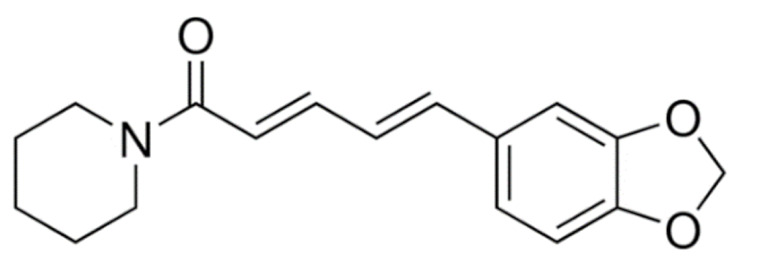	C_17_H_19_NO_3_	*Piper nigrum*	Anticancer
	Piperlongumine 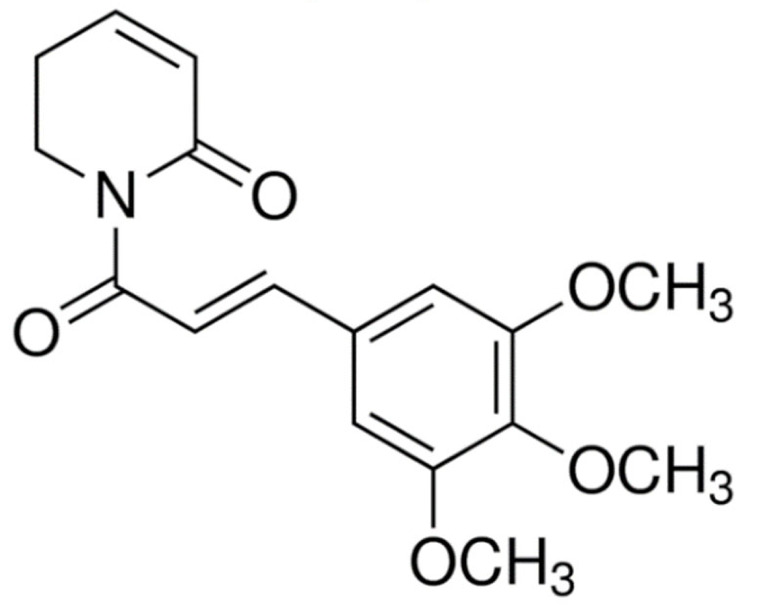	C_17_H_19_NO_5_	*Piper longum*	
	*Pipernonaline* 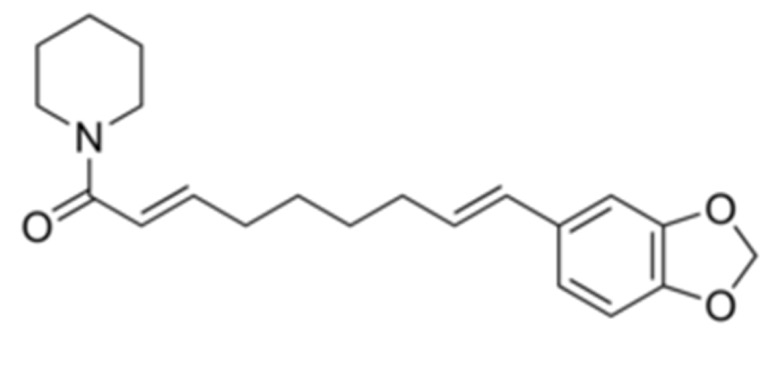	C_21_H_27_NO_3_		Antifungal
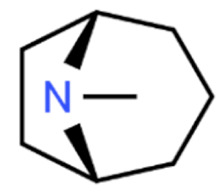 Tropanes	Atropine 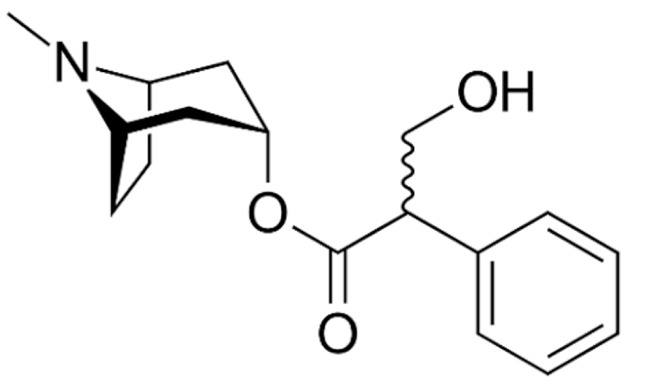	C_17_H_23_NO_3_	*Atropa belladonna*	Anticholinergic
	Cocaine 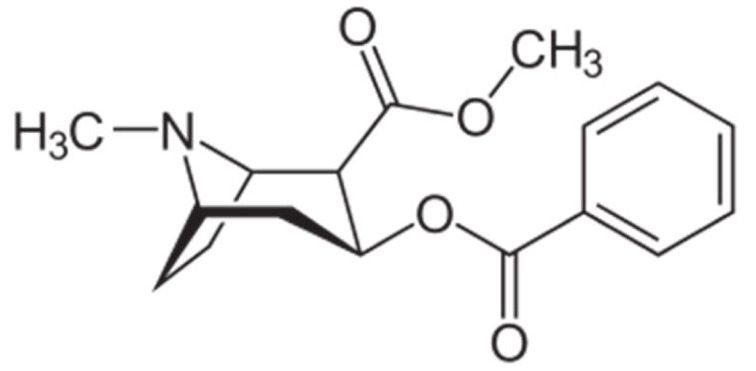	C_17_H_21_NO_4_	*Erythroxylum coca*	Local anaesthetic
	Hyoscyamine 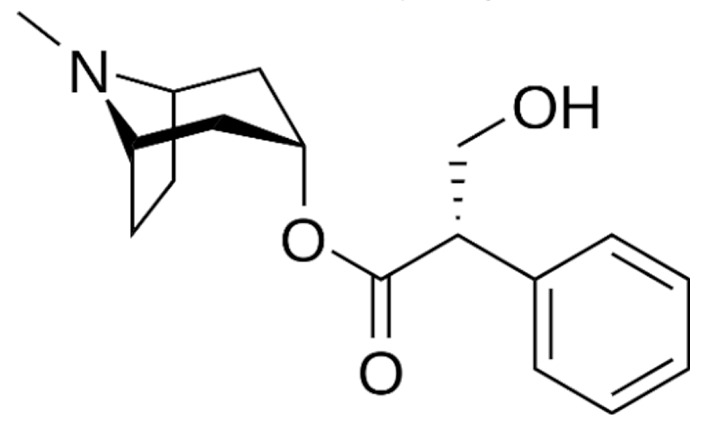	C_17_H_23_NO_3_	*Atropa belladonna, Hyoscyamus niger*	Anticholinergic
	Hyoscine 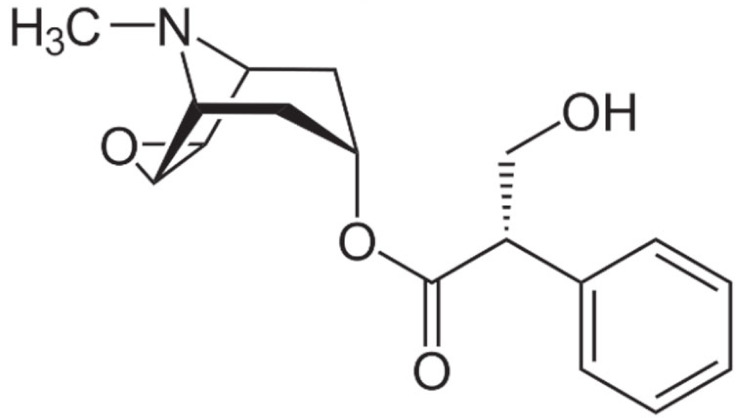	C_17_H_21_NO_4_	*Atropa belladonna*	Motion sickness
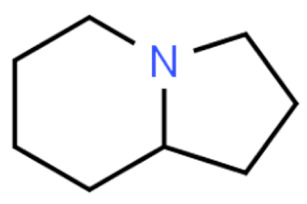 Indolizidine	Swainsonine 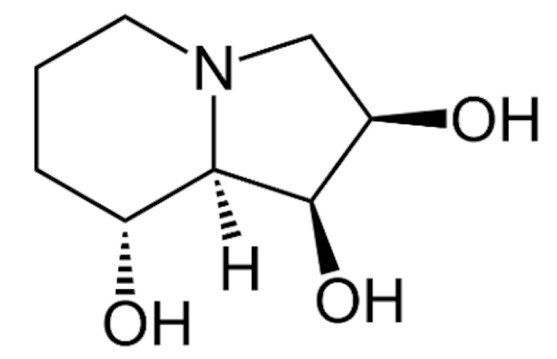	C_8_H_15_NO_3_	*Swainsona canescens*	Anticancer
	Castanospermine 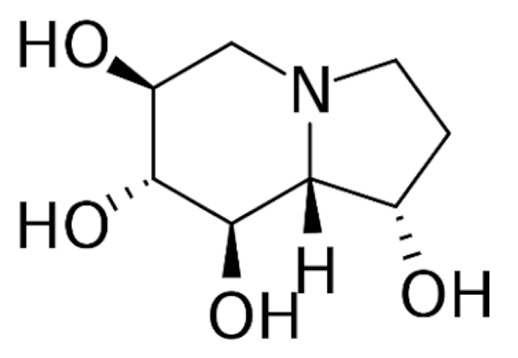	C_8_H_15_NO_4_	*Castanospermum australe*	Antiviral
	Securinine 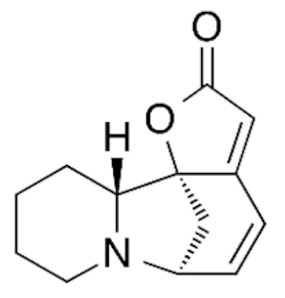	C_13_H_15_NO_2_	*Securinega suffruticosa*	Neuroprotection
	Tylophorine 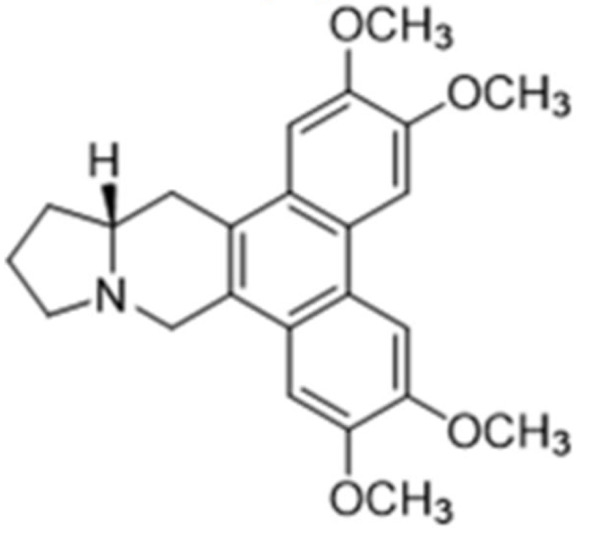	C_24_H_27_NO_4_	*Tylophora indica*	Anticancer
	Lycorine 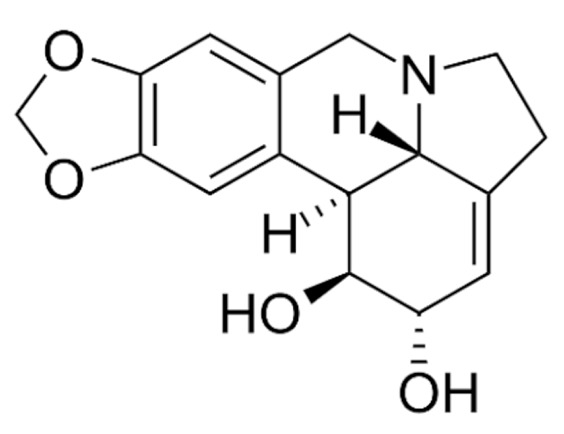	C_16_H_17_NO_4_	*Clivia miniata*	
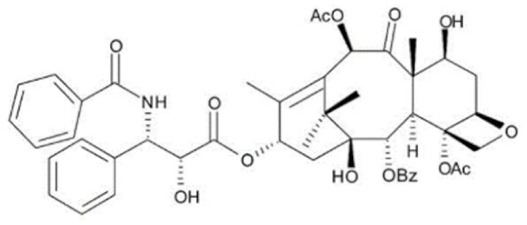 Terpenoids	Paclitaxel 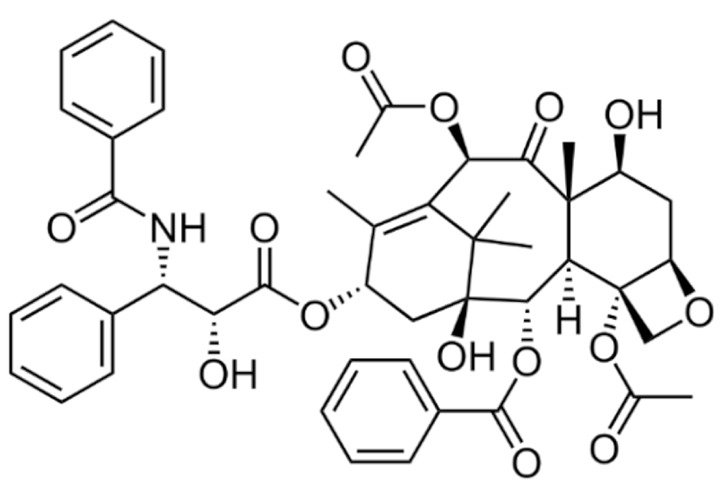	C_47_H_51_NO_14_	*Taxus brevifolia*	
	Docetaxel 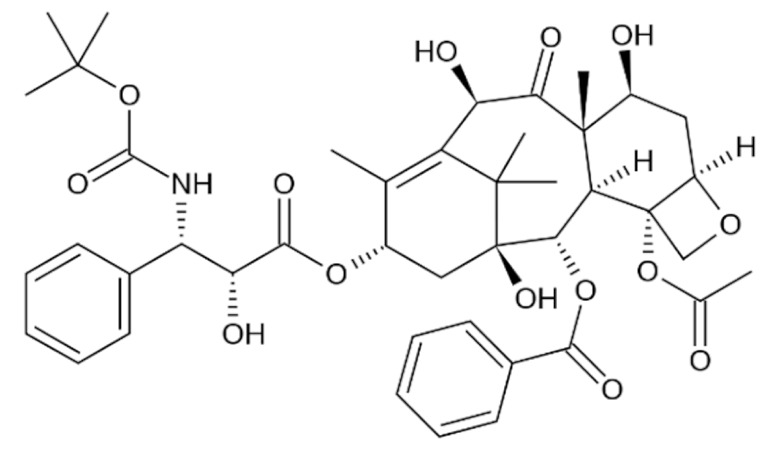	C_43_H_53_NO_14_	*Taxus baccata*	
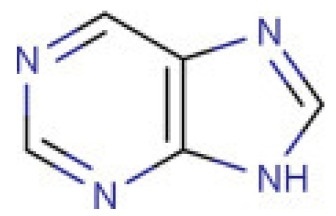 Purine	Caffeine 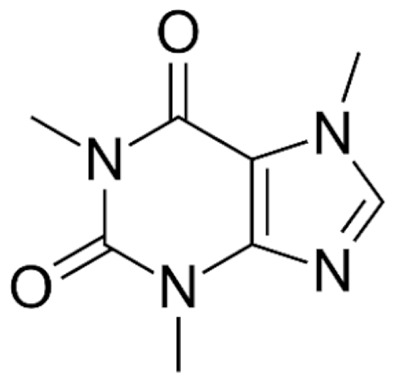	C_8_H_10_N_4_O_2_	*Coffee arabica*	CNS stimulant
	Theobromine 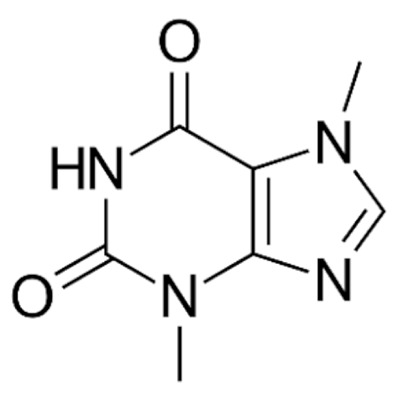	C_7_H_8_N_4_O_2_	*Theobroma cacao*	Cardioprotectant
	Theophylline 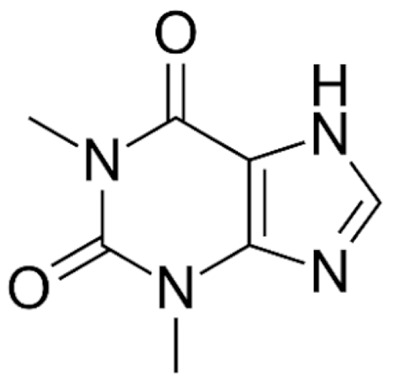	C_7_H_8_N_4_O_2_		COPD and asthma
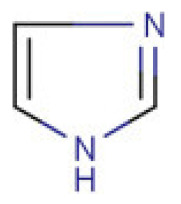 Imidazole	Pilocarpine 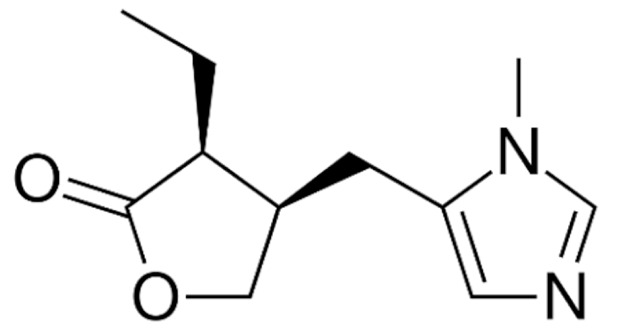	C_11_H_16_N_2_O_2_	*Pilocarpus microphyllus*	Glaucoma
	Epiisopiloturine 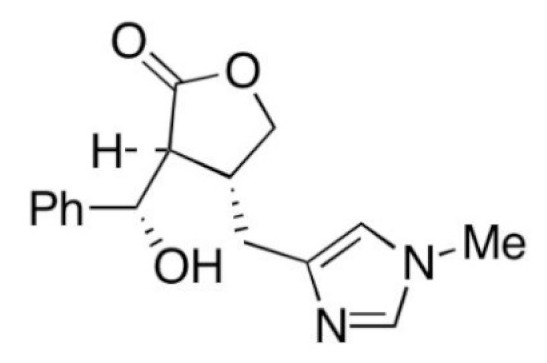	C₁₆H₁₈N₂O₃		Anthelmintic
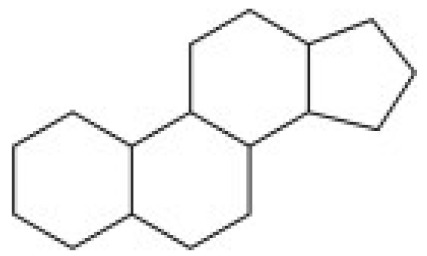 Steroidal	Pancuronium 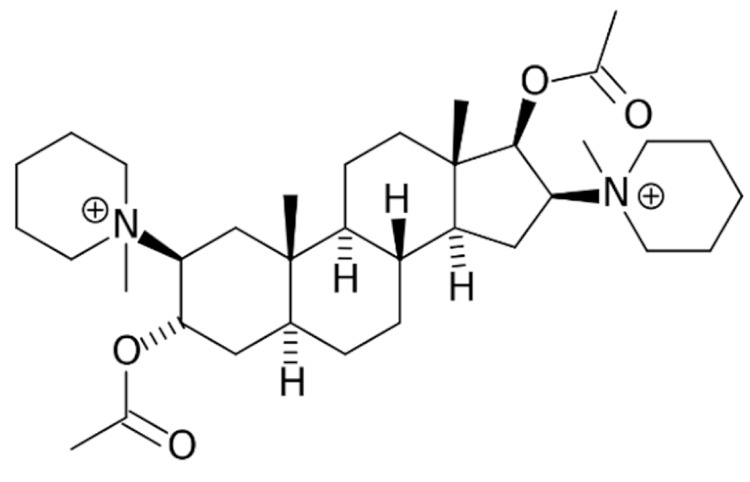	C_35_H_60_N_2_O_4_	*Malouetia bequaertiana*	Skeletal muscle relaxant
	Conessine 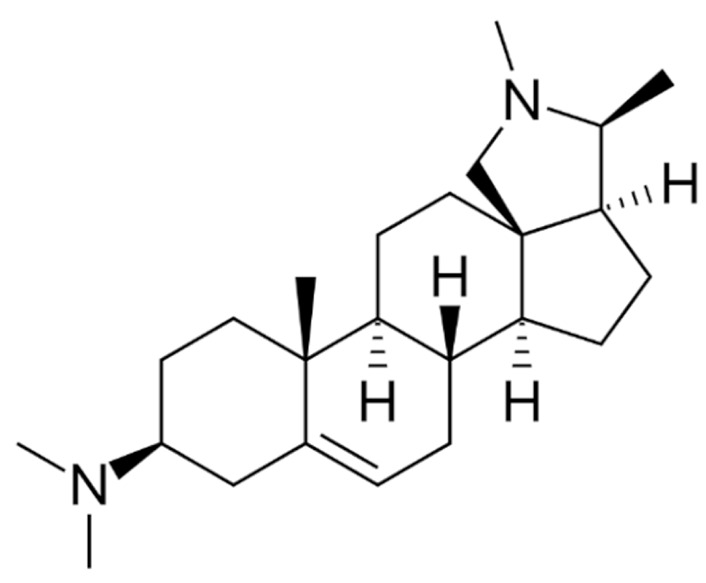	C_24_H_40_N_2_	*Holarrhena antidysenterica*	Antidysenteric
	Solanidine 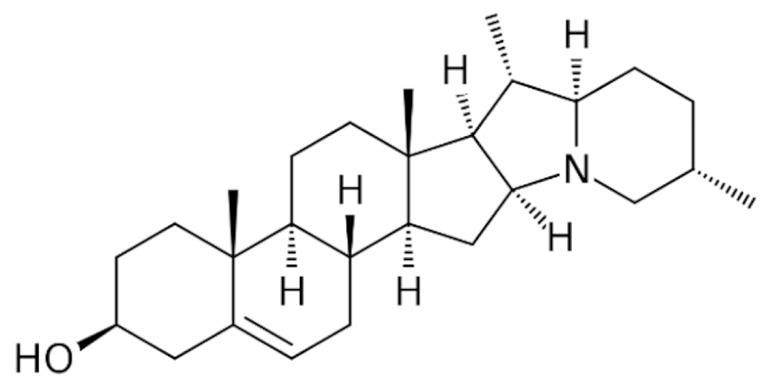	C_27_H_43_NO	*Solanum tuberosum*	Anticancer
	Tomatidine 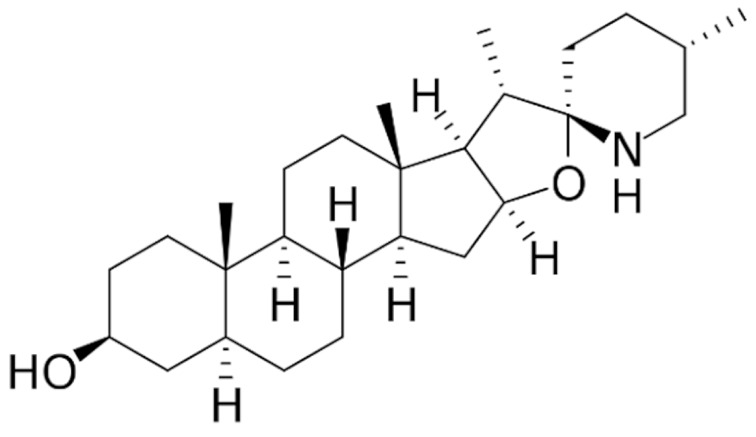	C_27_H_45_NO_2_	*Lycopersicon esculentum*	

**Table 2:** Alkaloids that have been Successfully Encapsulated in Lipid-based Nanoparticles.

Class	Compound	Type of Lipid Carrier	Type of Cancer	In Vitro/In Vivo	Cell Type/Animal Model	Ref.
Terpenoids	Docetaxel 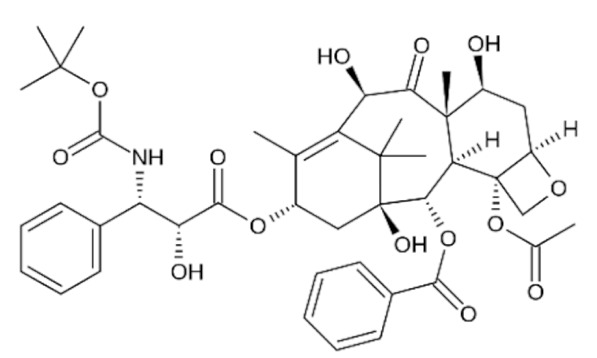	Liposome	Breast	in vitro; in vivo	MCF-7/4T1 xenograft mice	[243]
				in vivo	MDA-MB-435/LCC6 xenograft mice	[244]
				in vitro	MCF-7	[245]
			Lung	in vivo	A549 xenograft rat	[246]
				in vitro	A549	[247]
				in vitro	A549	[245]
			Liver	in vitro	HepG2	[245]
			Melanoma	in vitro; in vivo	B16F10/B16-F10 xenograft mice	[248]
		Micelle	Breast	in vitro	MCF-7	[249]
				in vitro	MCF-7	[250]
			Lung	in vitro	A549	[250]
		Niosome	Breast	in vitro; in vivo	MDA-MB-s31 and MCF-7	[251]
		NLC	Liver, Ovarian, Lung, Melanoma	in vitro; in vivo	HepG2, SKOV3, A549, B16 cells	[252]
	Paclitaxel 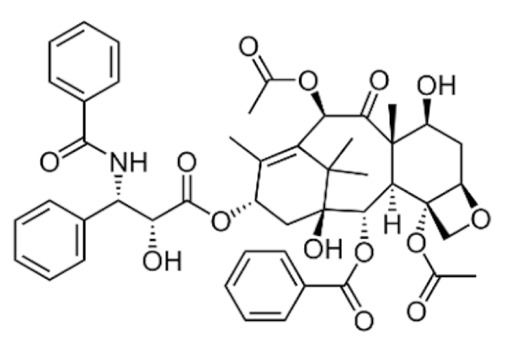	Liposome	Breast	in vitro; in vivo	4T1 xenograft mice	[253]
				in vivo	ICR male mice	[254]
			Lung	in vivo	A549 xenograft mice	[255]
				in vitro; in vivo	A549	[69]
		NLC	Breast, Ovarian	in vitro	MCF-7, SKOV3	[256]
		Ethosome	Squamous Cell Carcinoma	in vitro	DJM-1	[257]
		Micelle	Glioma	in vitro; in vivo	C6/C6 xenograft rat	[258]
Indole	Vincristine 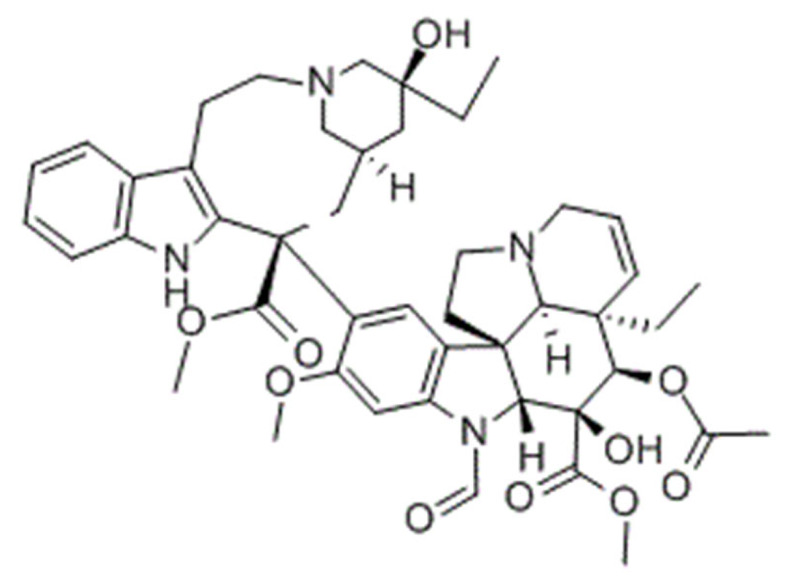	Liposome	Acute Lymphoblastic Leukemia	in vivo	Namwala xenograft mice	[190]
			Brain Glioma	in vitro, in vivo	Glioma bearing mice	[259]
			Nasopharyngeal cancer	in vitro, in vivo	KB/KBv200 xenograft mice	[260]
		SLN	Breast	in vitro; in vivo	MDA-MB-231	[261]
		NLC	Breast	in vitro	MCF-7	[262]
	Vinblastine 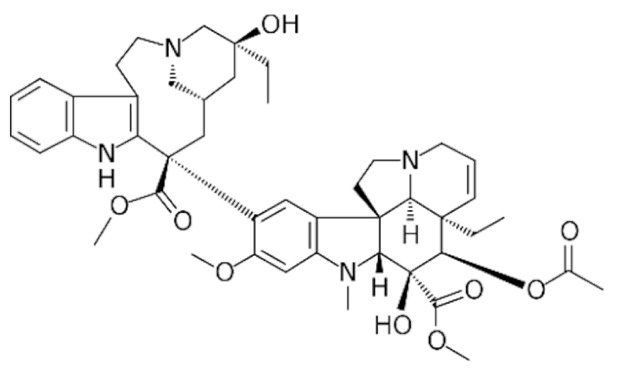	Liposome	Non-Small Cell Lung	in vitro; in vivo	LLT/LLT xenograft mice	[263]
		Niosome	Lung	in vitro; in vivo	TC-1/TC-1 xenograft mice	[264]
	Vinorelbine 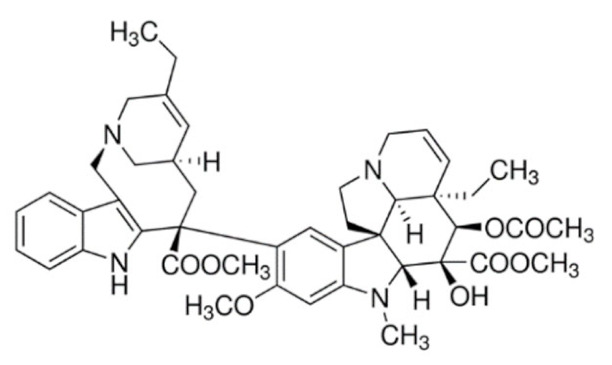	Micelle	Breast	in vitro	MCF-7	[265]
Quinoline	Topotecan 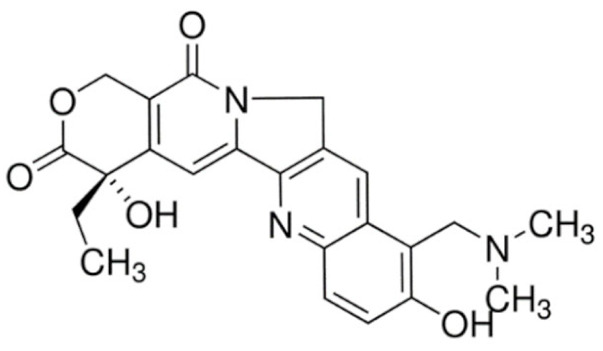	Liposome	Lung and Adenocarcinoma	in vitro	LLC	[266]
			Breast	in vitro; in vivo	MCF-7/MCF-7 xenograft mice	[267]
		SLN/NLC	Leukemia	in vitro	K-562	[268]
	Irinotecan 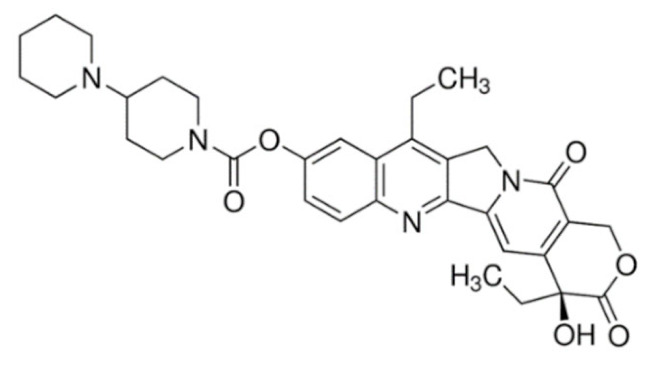	Liposome	Colon	in vivo	Colo 320DM and Colon 26 xenograft mice	[269]
		SLN	Rectal, Colon	in vivo	SCC7 xenograft mice	[270]
Isoquinoline	Berberine 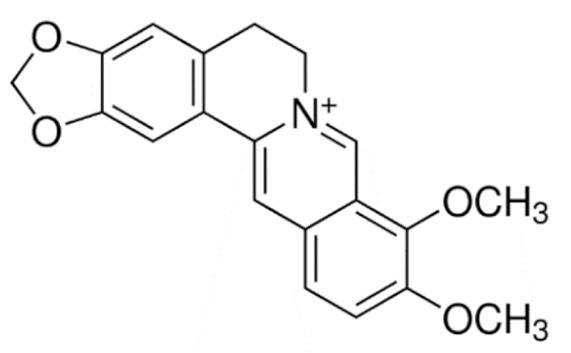	Liposome	Hepatic	in vitro; in vivo	HepG2/HepG2 xenograft mice	[271]
			Breast	in vitro; in vivo	MCF-7/MCF-7 CSC xenograft mice	[272]
		SLN	Breast, Hepatic, Lung	in vitro	MCF-7, HepG2, A549	[273]

**Table 3:** In vitro Efficacy of Alkaloids in Lipid-based Nanoparticles.

Type of Lipid Carrier	Alkaloids	Type of Cancer	Cell Line	IC_50_				Ref.
				Exposure Time (h)	Free Drug (μg/mL)	Drug-Carrier (μg/mL)	Reduction in IC_50_ (%)	
Liposome	Vincristine	Oral Epidermoid	KB	72	3.47	0.0021	99.94	[260]
		MDR Oral Epidermoid	KBv200	72	1.31	0.33	74.88	
	Topotecan	Breast	BT20	24	1.88	0.33	82.45	[266]
		MDR Breast	MCF-7/ADR	48	2.47	0.71	71.43	[267]
		Non-Small Cell Lung	LLC	24	2.49	0.78	68.67	[266]
		Breast	MCF-7	48	2.07	2.07	-0.22	[267]
	Docetaxel	Breast	MCF-7	72	14.40	1.90	86.81	[243]
		Non-Small Cell Lung	A549	24	9.39	2.42	74.23	[245]
		Hepatocellular	HepG2	24	7.91	3.12	60.56	[245]
		Breast	MCF-7	24	8.40	3.77	55.12	[245]
		Melanoma	B16F10	48	12.46	6.60	47.03	[248]
		Breast	4TI	72	13.00	8.70	33.08	[243]
		Breast	TUBO	72	4.70	4.10	12.77	[243]
	Berberine	Hepatocellular	HepG2	72	4.23	1.67	60.52	[271]
	Paclitaxel	Non-Small Cell Lung	A549	72	35.42	28.25	20.24	[69]
NLC	Vincristine	Breast	MCF-7	24	0.011	0.0031	71.97	[262]
		Diffuse large B-cell lymphoma	LY1	48	9.82	3.31	66.29	[277]
	Docetaxel	Non-Small Cell Lung	A549	96	0.60	0.12	79.73	[252]
		Ovarian	SKOV3	96	0.065	0.016	75.00	
		Hepatocellular	HepG2	96	0.78	0.38	51.04	
		Murine melanoma	B16	96	0.58	0.36	38.89	
	Paclitaxel	MDR Breast	MCF-7/ADR	48	8.61	0.065	99.25	[256]
		MDR Ovarian	SKOV3-TR30	48	9.35	0.10	98.93	
		Breast	MCF-7	48	0.29	0.075	74.14	
		Ovarian	SKOV3	48	0.16	0.053	66.88	
SLN	Berberine	Non-Small Cell Lung	A549	24	13.45	5.11	62.00	[273]
		Hepatocellular	HepG2	24	3.46	1.61	53.40	
		Breast	MCF-7	24	13.45	6.90	48.75	
	Vincristine	Breast	MDA-MB-231	72	0.09	0.078	11.05	[261]
Micelle	Docetaxel	Breast	MCF-7	72	9.00	0.090	99.00	[250]
		Non-Small Cell Lung	A549	72	12.80	0.56	95.63	
		Breast	MCF-7	48	1.28	0.25	80.39	[249]
	Vinorelbine	Breast	MCF-7	72	8.13	1.20	85.24	[265]
	Paclitaxel	Glioma	C6	48	0.36	0.24	32.92	[258]
Niosome	Docetaxel	Breast	MDA-MB-231	24	12.34	5.47	55.67	[251]
			MCF-7	24	0.72	0.51	28.95	
	Vinblastine	Lung	TC-1	72	7.40	13.30	-79.73	[264]

**Table 4:** In vivo Efficacy and Toxicity of Alkaloids in Lipid-based Nanoparticles.

Type of Lipid Carrier	Alkaloids	Type of Cancer	Animal Model	Cancer Cell	Route of Administration	Reduction in Tumor Volume (%)		Weight Changes (%)		Ref.
						Vs. Negative Control	Vs. Free Drug	Free Drug	Lipid-Carrier	
Liposome	Irinotecan	Colorectal Adenocarcinoma	Female ddY mice	Colo 320DM	IV	97	92.5	−18.1	−5.2	[269]
		Murine Colon	Female ddY mice	Colon 26	IV	36	14.7	+7.8	+3.9	
	Paclitaxel	Breast	BALB/c mice	4T1	IV	96.6	89.8	+8.6	+19	[253]
		Non-Small Cell Lung	Male BALB/c mice	A549	IV	55.6	23.8	+7.9	+6.4	[255]
		Breast	ICR male mice	-	IV	38.2	10.5	0	+30	[254]
	Docetaxel	Breast	Female BALB/c mice	TUBO	IV	85.7	83.3	+14	+22	[243]
				4T1	IV	78.9	71.4	+18	+36	
		Non-Small Cell Lung	Sprague- Dawley rats	A549	IV	96.3	50	−13	+5.6	[246]
		Breast	Female RAG2-M mice	MDA-MB-435/LCC6	IV	34.8	+25	NR	NR	[183]
		Melanoma	C57BL/6 mice	B16-F10	IV	12	1.8	−14.3	+14.3	[248]
	Topotecan	Breast	NCR nu/nu athymic female mice	BT474	IV	83.3	63.6	NR	NR	[284]
		MDR Breast	Female BALB/c nude mice	MCF-7/ADR	IV	75.3	58.2	NR	NR	[267]
	Vinblastine	Non-Small Cell Lung	C57BL/6 mice	LLT	IV	54.3	30.4	NR	NR	[263]
	Berberine	Breast	Female BALB/c nude mice	MCF-7 CSC	IV	47.4	23.1	NR	NR	[272]
	Vincristine	MDR Oral Epidermoid	Male nude mice	KBv200	IV	44.3	2.9	NR	NR	[260]
NLC	Docetaxel	Murine melanoma	Female Kunming Mice	B16	IV	62	32	+20	+33.3	[252]
	Vincristine	Diffuse Large B-Cell Lymphoma	BALB/c mice	LY-1	IV	44.2	21.6	−14.8	+19.1	[277]
SLN	Irinotecan	Squamous Cell	Female athymic nude mice	SCC7	Rectal	70	58.3	−6.9	+0.8	[270]
Niosome	Docetaxel	Breast	Female nude mice	MCF-7	PO	53.1	21.1	+26.3	+27	[251]
	Vinblastine	Murine Lung	Female C57BL/6 mice	TC-1	IV	33.3	18.5	NR	NR	[264]

Abbreviations: IV: Intravenous; PO: Oral.

**Table 5:** Clinically Approved Anticancer Alkaloids in Lipid-Based Nanoparticles and Clinical Trials to Date.

Alkaloids	Product Name	Type of Lipid-Based Nanoparticles	Indication	Status
Paclitaxel	Lipusu	Liposome	Squamous Non-small-cell Lung Cancer	Approved by State FDA of China (2006); Phase IV ongoing (NCT02996214)
	LEP-ETU	Liposome	Metastatic breast cancer	Phase II completed (NCT01190982)
	EndoTAG-1	Cationic Liposome	Locally advanced and/or metastatic adenocarcinoma of the pancreas	Phase III ongoing (NCT03126435)
Docetaxel	CPC634(CriPec docetaxel)	Micelle	Ovarian cancer	Phase II ongoing (NCT03742713)
Irinotecan	Onivyde	Liposome	Metastatic pancreatic cancer	Approved by FDA (2015)
			Small cell lung cancer	Phase III ongoing (NCT03088813)
	LY01610	Liposome	Small cell lung cancer	Phase II ongoing (NCT04381910)
			Advanced solid tumors	Phase I ongoing (NCT04088604)
Topotecan	FF-10850	Liposome	Advanced solid tumors	Phase I ongoing (NCT04047251)
	TLI	Liposome	Advanced solid tumors	Phase I ongoing (NCT00765973)
Lurtotecan	OSI-211	Liposome	Small cell lung cancer	Phase II completed (NCT00046787)
	NX-211	Liposome	Metastatic or locally recurrent head and neck cancer	Phase II completed (NCT00022594)
Vincristine	Marqibo (Onco TCS)	Liposome	Acute lymphoblastic leukemia	Approved by FDA (2012)
Vinorelbine	TLC178	Liposome	Advanced malignancies	Phase I/II ongoing (NCT02925000)

## Abbreviations

0-D	No Dimension
1-D	One Dimension
2-D	Two-Dimension
ABC	ATP-Binding Cassette
ABCB1	ABC Sub-Family B Member 1
ABCC1	ABC transporter C1
ABCG2	ABC transporter G2
ATM	Ataxia-Telangiectasia Mutated
ATR	Ataxia–Telangiectasia and RAD3-Related
AUC	Area Under The Curve
BAK	Bcl-2 Homologous Antagonist Killer
BAX	Bcl-2-Associated X Protein
BCL	B cell lymphoma
BCRP	Breast Cancer Resistance Protein
BH3	Bcl-2 Homology 3
BIND-014	PSMA-Targeted Docetaxel Nanoparticles
CDK	Cyclin-Dependent Kinase
Chk2	Checkpoint Kinase 2
CL	Clearance
C_max_	Peak Drug Concentration
COVID-19	Coronavirus Disease 2019
CR	Complete Remission
CYP	Cytochrome P450
DDR	DNA Damage Response
DISC	Death-Inducing Signaling Complex
DNA	Deoxyribonucleic Acid
DR3	Death Receptor 3
EGFR	Epidermal Growth Factor Receptor
EPR	Enhanced Permeability and Retention
FADD	Fas-Associated Protein With Death Domain
FDA	Food and Drug Administration
GDP	Guanosine 5′-Diphosphate
GTP	Guanosine 5′-Triphosphate
IC50	Half-Maximal Inhibitory Concentration
ID	Injected Dose
ILs	Interleukins
Lys	Lysine
MCL-1	Myeloid Cell Leukemia 1
MDR	Multidrug Resistance
MOMP	Mitochondria Outer Membrane Permeabilization
MRD	Minimal Residual Disease
MRE11	Meiotic Recombination 11 Homolog 1
MRI	Magnetic Resonance Imaging
MRN	MRE11–RAD50–NBS1
mRNA	Messenger Ribonucleic Acid
MTD	Maximum Tolerated Dose
NLC	Nanostructured Lipid Carriers
NNI	National Nanotechnology Initiative
Orn	Ornithine
pCR	Pathological Complete Response
PEG	Polyethylene Glycol
Phe	Phenylalanine
PSMA	Prostate Specific Membrane Antigen
RAD50	Double Strand Break Repair Protein
RB	Retinoblastoma
RES	Reticuloendothelial System
SAC	Spindle Assembly Checkpoint
SARS-CoV-2	Severe Acute Respiratory Syndrome Coronavirus 2
scFv	Single Chain Fragment Variable
SCLC	Small Cell Lung Cancer
SGT-53	Tumor Suppressor Gene TP53/scL-p53
SLN	Solid Lipid Nanoparticles
SN-38	7-Ethyl-10-Hydroxycamptothecin
SN-38G	SN-38 Glucuronide
*t*_1/2_	Elimination half-life
TfR	Transferrin Receptor
TL1A	TNF-Like Cytokine 1A
TNF	Tumor Necrosis Factor
TOP1cc	Topoisomerase I-DNA Cleaved Complexes
Trp	Tryptophan
Tyr	Tyrosine
UGT1A	Uridine Diphosphate Glucuronosyltransferase 1A
V_d_	Volume of Distribution
VYXEOS	Liposomal Cytarabine/Daunorubicin
